# Peste des Petits Ruminants at the Wildlife–Livestock Interface in the Northern Albertine Rift and Nile Basin, East Africa

**DOI:** 10.3390/v12030293

**Published:** 2020-03-07

**Authors:** Xavier Fernandez Aguilar, Mana Mahapatra, Mattia Begovoeva, Gladys Kalema-Zikusoka, Margaret Driciru, Chrisostom Ayebazibwe, David Solomon Adwok, Michael Kock, Jean-Paul Kabemba Lukusa, Jesus Muro, Ignasi Marco, Andreu Colom-Cadena, Johan Espunyes, Natascha Meunier, Oscar Cabezón, Alexandre Caron, Arnaud Bataille, Genevieve Libeau, Krupali Parekh, Satya Parida, Richard Kock

**Affiliations:** 1 Department of Pathobiology and Population Sciences, Royal Veterinary College, London NW1 0TU, UK, mattia.begovoeva@edu.unito.it (M.B.); nmeunier@animalhealthireland.ie (N.M.); rkock@rvc.ac.uk (R.K.); 2 Department of Ecosystem and Public Health, Faculty of Veterinary Medicine, University of Calgary, 3280 Hospital Dr. NW, Calgary, AB T2N 4Z6, Canada; 3 The Pirbright Institute, Ash Road, Pirbright, Woking, Surrey GU24 0NF, UK, mana.mahapatra@pirbright.ac.uk (M.M.); krupali.parekh@pirbright.ac.uk (K.P.); satya.parida@pirbright.ac.uk (S.P.); 4 Dipartimento di Scienze Veterinarie, Università degli Studi di Torino, Largo Paolo Braccini 2, 10095 Grugliasco, Italy; 5 Conservation Through Public Health, Plot 3 Mapera Lane, Uring Crescent, P.O. Box 75298 Entebbe, Uganda; gladys@ctph.org; 6 Uganda Wildlife Authority (UWA), Plot 7 Kira Road, P.O. Box 3530 Kampala, Uganda; margaret.driciru@wildlife.go.ug; 7 NADDEC Ministry of Agriculture, Animal Industries and Fisheries, P.O. Box 102 Entebbe, Uganda; cayebazibwe@gmail.com; 8 Central Veterinary Diagnostic Laboratories, Ministry of Animal Resources and Fisheries, P.O. Box 126 Juba, South Sudan; davidojwok@yahoo.com; 9 Consultant Field Veterinary Programme, Formerly: Wildlife Conservation Society, 2300 Southern Boulevard Bronx, NY 10460, USA; mdkock@kingsley.co.za; 10 Regional Gorilla Conservation Employees Health Program, MGVP Inc., Goma 00243, Democratic Republic of the Congo; jp.lukusa@gmail.com; 11 Daktari, La Solana 35, AD700 Escaldes, Andorra; jmurofigueres@gmail.com; 12 Servei d’Ecopatologia de Fauna Salvatge (Sefas) and Wildlife Conservation Medicine Research Group (WildCoM), Departament de Medicina i Cirurgia Animals, Universitat Autònoma de Barcelona, 08193 Bellaterra, Spain; ignasi.marco@uab.cat (I.M.); andreuccadena@gmail.com (A.C.-C.); johan.espunyes@gmail.com (J.E.);; 13 Research and Conservation Department, Zoo de Barcelona. Parc de la Ciutadella s/n, 08003 Barcelona, Spain; 14 UAB, Centre de Recerca en Sanitat Animal (CReSA, IRTA-UAB), Campus de la Universitat Autònoma de Barcelona, 08193 Bellaterra, Spain; 15 CIRAD, UMR ASTRE, F–34398 Montpellier, France; alexandre.caron@cirad.fr (A.C.); arnaud.bataille@cirad.fr (A.B.); genevieve.libeau@cirad.fr (G.L.); 16 ASTRE, Univ Montpellier, CIRAD, INRAE, F-34398 Montpellier, France; 17 Veterinary Faculty, Eduardo Mondlane University, Maputo 1102, Mozambique

**Keywords:** peste des petits ruminants, PPR, wildlife, Uganda, South Sudan, Democratic Republic of the Congo, transboundary emerging diseases, epidemiology, host range

## Abstract

In the recent past, peste des petits ruminants (PPR) emerged in East Africa causing outbreaks in small livestock across different countries, with evidences of spillover to wildlife. In order to understand better PPR at the wildlife–livestock interface, we investigated patterns of peste des petits ruminants virus (PPRV) exposure, disease outbreaks, and viral sequences in the northern Albertine Rift. PPRV antibodies indicated a widespread exposure in apparently healthy wildlife from South Sudan (2013) and Uganda (2015, 2017). African buffaloes and Uganda kobs <1-year-old from Queen Elizabeth National Park (2015) had antibodies against PPRV N-antigen and local serosurvey captured a subsequent spread of PPRV in livestock. Outbreaks with PPR-like syndrome in sheep and goats were recorded around the Greater Virunga Landscape in Kasese (2016), Kisoro and Kabale (2017) from western Uganda, and in North Kivu (2017) from eastern Democratic Republic of the Congo (DRC). This landscape would not be considered typical for PPR persistence as it is a mixed forest–savannah ecosystem with mostly sedentary livestock. PPRV sequences from DRC (2017) were identical to strains from Burundi (2018) and confirmed a transboundary spread of PPRV. Our results indicate an epidemiological linkage between epizootic cycles in livestock and exposure in wildlife, denoting the importance of PPR surveillance on wild artiodactyls for both conservation and eradication programs.

## 1. Introduction

Peste des petits ruminants virus (PPRV) is a small ruminant morbillivirus from the Paramyxoviridae family that groups with other notorious members like Rinderpest virus (RPV), measles virus, canine, or phocine distemper virus [1]. The disease peste des petits ruminants (PPR), is one of the most important and devastating infectious diseases in domestic small ruminants across more than 70 countries, causing economic losses of 1.45 to 2.1 billion United States Dollar (USD) each year, potentially threatening 80% of the worldwide small livestock and the livelihoods of 900 million poor farmers [2,3]. PPR is also a significant disease and the cause of mortality in captive and wild artiodactyls in Asia and the Middle East, and an emerging threat to wildlife conservation [4,5,6]. 

Clinical signs of PPR are associated to the lymphotropic and epitheliotropic nature of the virus and comprise of depression, conjunctivitis, ocular and nasal discharge, erosions/ulcers of the oral mucosa, respiratory distress, and diarrhea and may eventually lead to death [7,8,9,10]. The peracute and acute syndrome in sheep and goats can cause nearly 100% of morbidity and high fatality rate [11]. However, both clinical signs and mortality can vary widely depending on viral strains, breeds, coinfections, and general nutrition and fitness. During the last decade, PPR experienced a dramatic geographic expansion to East Asia and re-emerged in endemic areas [1,7,12]. Following previous successful experiences with Rinderpest (RP) eradication, the World Organization for Animal Health (OIE) and the United Nations Food and Agriculture Organization (FAO) launched an eradication program for PPR that aims to control and eradicate the disease by 2030 [13]. A significant constraint on reaching this goal is the lack of understanding of the PPR epidemiology at the wildlife–livestock interface, in which several domestic and wild species may be involved [5,14].

One of the more diverse and complex domestic and wild ungulate communities occurs in East Africa. The first PPR cases described in livestock from East Africa date from 1971–1972 in Sudan [15], followed by outbreaks confirmed in Ethiopia in 1989–1990 [16]. Despite some serological evidences of PPRV incursions into Uganda and Kenya in the 1980s [17] and early 2000s [18], and an isolated report of an outbreak in Uganda in 2003 [19], it was not until 2006–2008 when the first official and large PPR outbreak was reported in Kenya and the bordering Karamoja region in Uganda, with further disease records in northern Tanzania (2007–2008) [18,20,21]. Since then, several PPR outbreaks have been described in different regions of East Africa, suggesting PPRV persistence in some nomadic and semi-nomadic pastoralist systems of South Sudan, north-eastern Uganda, north-western Kenya, and northern Tanzania (Table S1) [22,23,24,25]. PPR appeared to be absent in Ugandan wildlife prior to 2004 from serosurveillance under the Pan African Rinderpest Campaign (PARC) and the Pan African Programme for the Control of Epizootics (PACE) for RP eradication, but African buffalo (*Syncerus caffer*) turned seropositive in analyses of convenience samples from 2004–2005 in different national parks across northern and western Uganda (Table S2), corresponding with the first reports of PPR in livestock in this country (Table S1). All the evidence suggests that PPRV is present in some parts of Uganda and since 2004 has caused outbreaks of disease in livestock when introduced to naïve herds and areas. A more detailed history of PPR in East Africa is provided as Supplementary Material, as well as a compilation of historical PPR records in Uganda, South Sudan, and Democratic Republic of the Congo (DRC) in Table S1.

The impact that PPR can have in wild animal populations is exemplified by the massive die-offs reported in wildlife from Mongolia [5] and mountain ungulates from the Middle East, and south and east of Asia [26,27,28,29]. African wildlife appears to also be commonly exposed to PPRV according to antibody and virus detection, but as of yet, there is little evidence of the disease in free-ranging populations [14,30,31]. The only recent credible report of PPR disease in free-ranging African wildlife was in a population of dorcas gazelles (*Gazella dorcas*) in Dinder National Park in Sudan in 2017, but epidemiological and pathological information are lacking in these reports [32]. A broad wild-domestic interface exists around unfenced natural areas and effective contacts for pathogen transmission increasingly occur between livestock and wildlife [33,34]. In fact, PPRV spillover from a domestic source was suggested in the Serengeti ecosystem in Tanzania with higher antibody prevalence in wildlife close to livestock, but without evident clinical syndromes or mortality [14]. Although it is becoming clearer as to the role of wildlife in PPR epidemiology, with most data supporting the hypothesis of wildlife as a victim rather than reservoir [5,14,27], there are still gaps in understanding. The circulation of PPRV among wildlife even on a temporary basis can contribute to the virus persistence in multi-host systems and enhance the spread of the virus [35]. Whether PPRV can be maintained in natural systems or at the wildlife–livestock interface is not known, but the high host plasticity exhibited by the virus poses a challenge for PPR control and eradication programs, as well as a continuous threat of disease outbreaks in vulnerable African wildlife.

One of the objectives of the Improved Understanding of the Epidemiology of Peste des Petits Ruminants (IUEPPR) project (ANIHWA ERANET BB/L013592/1 and BB/L013657/1) was to study the status of PPR in wildlife from eastern Africa and explore the epidemiological links with ongoing livestock cycles. Under that framework, this study aims to provide a perspective on PPR of the northern region of the Albertine Rift as a whole, over a recent time period, and more specifically study PPR at the wildlife–livestock interface in western Uganda, which represents a savannah–forest transition ecozone with relatively low interactions of small livestock with wildlife. The PPRV strains recovered opportunistically during the study period from across the Ugandan border in the Democratic Republic of the Congo were also studied.

## 2. Materials and Methods

This purposive epidemiological study was designed to detect the presence of disease through observation and/or detection of infection by serology in sampled African buffalo and Uganda kob (*Kobus kob thomasi*) in Queen Elizabeth National Park (QENP). Other opportunistic sampling, for other purposes, in western Uganda from livestock in communities around QENP, wildlife from South Sudan, and suspected PPR infected flocks of small domestic ruminants in DRC, provided context for the wildlife serological results in the region.

### 2.1. Study Areas

The northern Albertine Rift borders with South Sudan and covers western Uganda, northeastern DRC, and Rwanda. It is one of the most biodiverse regions of Africa and comprises large water bodies and un-fenced natural areas, composed by a mosaic of grasslands, woodland savanna, and dense tropical forests [36]. This natural heritage value is recognized by several protected areas that are transboundary and compose the Greater Virunga Landscape, including QENP, Mgahinga Gorilla National Park (MGNP), and Virunga National Park. The Albertine Rift is also an area with relatively large and dense human populations of mainly sedentary agro-pastoral systems [37]. Further characteristics of natural and livestock systems in study areas are provided by country as Supplementary Material. 

### 2.2. Sampling and Data

#### 2.2.1. Western Uganda

For the detection of PPRV antibodies in wildlife from QENP, a minimum sampling size of 39, with 3 animals per herd, was calculated assuming an expected herd prevalence of 20% and a within-herd prevalence of 60% (95% confidence, 90% of test sensitivity), taking into account that previous surveys on PPR reported 25%–100% seroprevalence in African buffalo and antelopes from East Africa [14,31,38]. Buffalo were captured from six different areas within QENP in 2015 and five areas in 2017, and Uganda kob from five mating grounds in 2015. Animals were selected as a convenience sample within herds among different age categories from > 6 months old. 

Most of the Uganda kob were captured by using a net system and immediately sedated, and buffalo and a few Uganda kob were chemically immobilized by using a dart gun from a vehicle. The drugs used for the wildlife captures are described in Table S3. Blood was collected by jugular or coccygeal venipuncture in buffalo and jugular venipuncture in kob. Clinical signs, sex, estimation of age, body condition, herd size, species composition, proximity to livestock, and location were recorded amongst other metadata.

**Figure 1:**
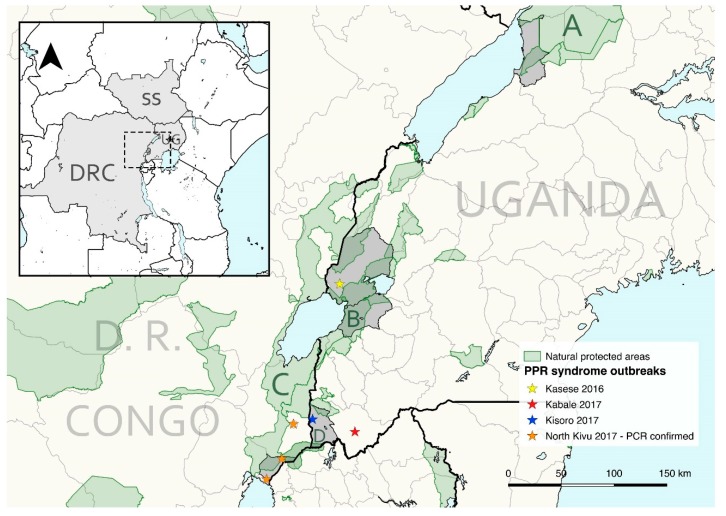
General map of East Africa (left upper corner) highlighting the countries included in this study: South Sudan (SS), Uganda (UG), and Democratic Republic of the Congo (DRC). The northern area of the Albertine Rift is magnified showing, shaded in grey, the administrative subdivisions of Uganda and DRC where livestock sampling was performed (from north to south: Buliisa district, Kasese district, Rubirizi district, Kisoro District, and Nyiragongo territory in North Kivu) and PPR-like outbreaks recorded in small livestock 2015–2017 reported in this study. The transboundary Greater Virunga Landscape includes the study areas of Queen Elizabeth National Park (B) and Mgahinga Gorilla National Park (D) in Uganda, and Virunga National Park in DRC (C). Murchison Falls Conservation Area is highlighted (A).

An opportunistic blood sampling of goats, sheep, and cattle was also performed in villages and communities bordering MFNP, QENP, and MGNP between 2015 and 2017, with a median distance of 1352 m (0–9153 m) to the natural protected areas. Sampled localities were Buliisa and Butiaba sub-counties from Buliisa district (MFNP), Karusandara, Katwe-Kabatoro, Kisinga, Kitswamba, Lake Katwe (including Katunguru at Kasenyi sector), Muhokya, Mukunyu, and Nyakatonzi from Kasese district (QENP), Katanda, Kicwamba, Kirugu, and Ryeru from Rubirizi district (QENP), and Muramba and Nyarusiza from Kisoro district (MGNP) (Figure 1 and Figure 3).

Cattle, sheep, and goats were sampled in aggregation or meeting points around the communities, and numbers depended on the herd size (10%–100% of the herd). The number of herds (owners) and herd size was mainly recorded in 2015 and 2017 and varied with a minimum of 61 herds and a maximum of 178 herds per species and year (Table S4). Blood was collected from the coccygeal vein in cattle and from the jugular vein in sheep and goats. 

#### 2.2.2. South Sudan 

Sera of wildlife from South Sudan were obtained opportunistically from captured animals between July and August 2013 from the Nile basin to Boma National Park (*n* = 87). These captures were performed to deploy radio-collars for conservation monitoring studies and included tiang (*Damaliscus lunatus tiang*), white eared kobs (*Kobus kob leucotis*), elands (*Taurotragus oryx*), and elephants (*Loxodonta africana*). Animals were darted from a helicopter platform using a Dan-Inject JM special dart gun and 1.8 mL darts with 2.0 × 35–40 mm collared needles (DAN-INJECT ApS, Børkop, Denmark) or a Dan-Inject gun with 13 mm barrel and 1–2 mL darts (Pneu-Dart, Inc., Williamsport PA, USA). The drugs used for chemical immobilization and its reversal are described in Table S3.

#### 2.2.3. Democratic Republic of the Congo

A suspicion of PPR in sheep and goats, based on clinical signs, was reported to Virunga National Park and followed up by park staff on 20 October 2017. The location was at Kibumba (1.48247° S, 29.34605° E), a settlement near to the Rwandan border and next to the park where buffalo regularly share pasture with local livestock (Figure 1). This was reported to the provincial agricultural department in North Kivu and samples were taken to the Goma Veterinary Laboratory including serum and nasal swabs from 14 sheep and goats and some organs after necropsy. A large number of sheep and goats died in Kibumba, Rutshuru, Kiwandja, and Goma, but no cases in wildlife were reported at the time by the Virunga National Park staff. The swab samples were subsequently shipped by the IUEPPR project to the project partner at The Pirbright Institute, UK for diagnostics and molecular characterization of the virus. 

### 2.3. Laboratory analyses

All blood tubes were centrifuged within 24 h post collection at 1200× *g* for 15 min and sera stored at –20 °C within 24 h of collection. Sera samples in remote areas were placed in a nitrogen liquid tank until arrival to central facilities in the country concerned and finally stored at –20 °C. 

The serum samples from Uganda were tested at The Pirbright Institute, UK using the N based competitive ELISA (IDVET, Montpellier, France) for detection of PPRV antibodies [39]. All wildlife and 317 livestock sera were analyzed in duplicate and the average of two results was used to determine the individual inhibition percentage. Wildlife sera from South Sudan were tested using the same cELISA kit at the Central Veterinary Diagnostics Laboratories from the Ministry of Animal Resources and Fisheries in Juba, South Sudan. Doubtful results with percentage inhibition values between 50% and 60% were considered negative. 

All 14 nasal swabs from the outbreak near to Virunga National Park in DRC were screened for the presence of viral nucleic acid by real-time reverse transcription-polymerase chain reaction (RT-qPCR). The swab samples were processed and total RNA extracted following a method described previously [40]. The extracted RNA was used in RT-qPCR and the assay was performed following the method as described by Batten and colleagues [41]. In addition, total RNA was also extracted from a tissue culture grown virus PPRV/Morocco/2008 [42], and this RNA was used as the positive control in all the molecular assays. The RNA from RT-qPCR positive samples were reverse transcribed and the C-terminus of the N-gene was amplified as previously described [43] using the superscript III One-Step RT-PCR kit (Invitrogen, Carlsbad, CA, USA). The PCR amplicons were purified using the GE Healthcare Illustra GFXPCR purification kit (GE Healthcare, Buckinghamshire, UK) according to the manufacturer’s instructions and sequenced using BigDye^®^ Terminator v3.1 Cycle Sequencing Kit (Applied Biosystems, Carlsbad, CA, USA) on an ABI 3730 machine. Sequences were assembled and analyzed using SeqMan pro (DNAStar Lasergene 13.0). The sequences obtained were deposited in Genbank under the accession numbers MT154039 (PPRV/DRC/01/2017) and MT154040 (PPRV/DRC/14/2017).

The partial N-gene sequences (255 nucleotides) available in GenBank for Africa including DRC, Uganda, and Burundi (*n* = 33) until November 2019 were retrieved and used for constructing a neighborhood-joining phylogenetic tree. Alignments of the N-gene sequences were made using the Clustal W program and used for construction of distance matrices using the Kimura 2-parameter nucleotide substitution model [44], as implemented in the program MEGA 6.0 [45]. A maximum-likelihood phylogenetic tree was then generated using MEGA 6.0, and the robustness of tree topology was assessed using 1000 bootstrap replicates.

### 2.4. Data analyses

Differences among sample prevalence from different years and species were assessed fitting generalized linear models with a logit link function, using the status on PPR antibodies with a binomial distribution as a response variable and setting significance at 0.05. The prevalence of antibodies in livestock species was estimated using sensitivity and specificity of the test, and calculated together with confidence intervals using the EpiR package [46]. All data analyses and graphs (ggplot2) were performed with R statistical software [47]. The maps and distance of sampling points to protected areas from western Uganda was done with QGIS [48].

## 3. Results

**Figure 2:**
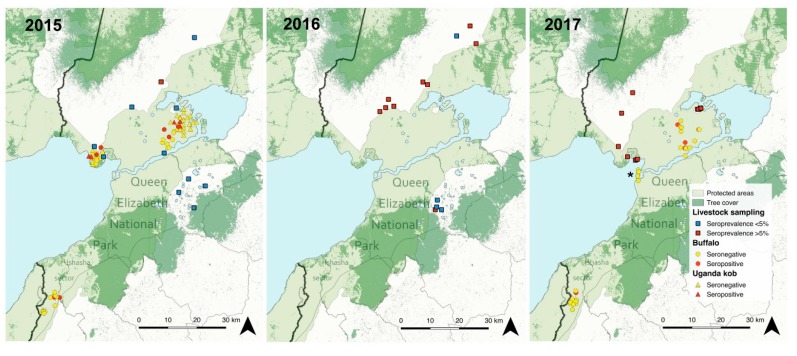
Sampling locations and results of PPRV antibody detection in livestock and wildlife from Queen Elizabeth National Park (QENP), western Uganda from 2015–2017. Livestock sampling locations with seropositive animals are shown. District borders are shown in grey and the national border with DRC is shown with a thicker black line. The asterisk indicates Mweya Peninsula in the right image in 2017. The Kazinga channel connects Lake Edward (left) with Lake George (right) and divides QENP. The tree cover within QENP indicates the location of Maramagambo Forest.

Captured African buffalo and Uganda kob did not have clinical signs, but antibodies against PPRV were detected in 2015 and 2017 in most of the areas sampled from QENP, with the exception of Keymale in the most southern part of the Ishasha sector and Mweya peninsula (Table 1; Figure 2). The overall prevalence of PPRV antibodies in buffalo was 19.0% (CI 95%, 10.9–30.8) including both years and 10.3% (CI 95%, 4.1–23.6) for Uganda kob, with an overall sample prevalence in Ugandan wildlife of 15.5% (CI 95%, 9.6–24.0) (Table 2). No statistical differences of seroprevalence in both individuals and herds were detected between years. Within herd antibody prevalence ranged from 25.0% to 66.7% in kob (*n* = 6 herds) and was 33.3% in buffalo (*n* = 8 herds); this was calculated only in herds with positive animals and with a minimum of three individuals sampled. 

**Figure 3:**
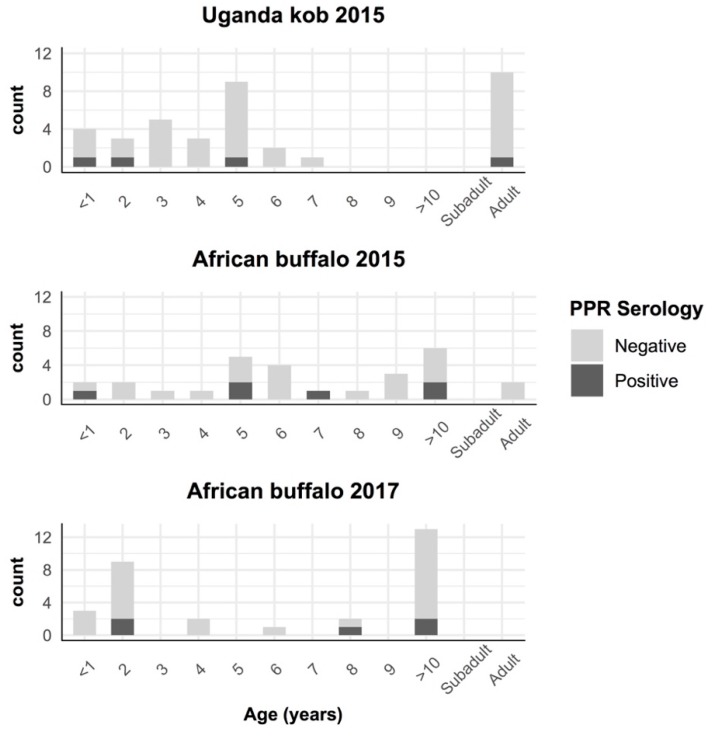
Results of PPRV antibody detection by age in African buffalo and Uganda kob captured in Queen Elizabeth National Park, Uganda. Animals without aging in years are classified as subadult (<3 years) and adult (>3 years).

There were seropositive buffalo and kob in all age groups from 2015, including buffalo and kob <1 year old. The youngest positive buffalo from 2017 was 2 years old (Figure 3). During the study period, no evidence of mortality events or PPR-like clinical syndromes were noted in wildlife from QENP by the Uganda Wildlife Authority.

Wildlife from South Sudan had antibodies against PPRV with an overall prevalence of 18.4% (CI 95%, 11.6–27.8). Almost all seropositive animals were tiang (*n* = 21) with a sample prevalence of 71.4% (CI 95%, 50.0–86.2), and one seropositive elephant (Table 1).

Including all the areas sampled, PPRV antibodies were found in wild animals from the families Bovidae and Elephantidae, involving members of the Alcelaphinae, Bovinae, Elephantinae, and Reduncinae subfamilies (Table 1). This is the first report of PPRV antibodies in tiang and elephant.

**Figure 4:**
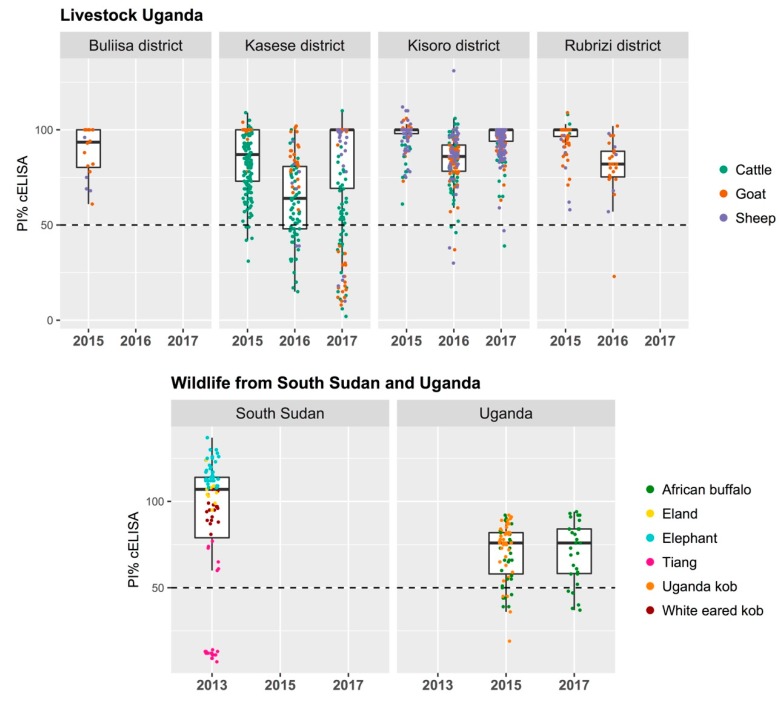
Percentage inhibition (PI) value distribution of peste des petits ruminants (PPR) N antigen-based competitive ELISA (IDVET, Montpellier, France) shown by species and locations sampled in Uganda and South Sudan. Wildlife from Uganda were sampled in Queen Elizabeth National Park and wildlife from South Sudan within the Nile basin and Boma National Park. Positive samples below the 50% percentage of inhibition are shown in the graphs with a black dashed line.

All livestock sampled in 2015 from Buliisa, Kasese, Rubirizi, and Kisoro districts from western Uganda were negative for the detection of PPRV antibodies with the exception of a few cattle in Kasese district (5/152) (Table 2). In 2016, a few sheep and goats resulted positive in Kasese (positive/total, 1/29), Rubirizi (1/26), and Kisoro districts (3/99), and there was significantly higher (*p* < 0.01) seroprevalence in cattle from Kasese as compared to small livestock (27/60). A similar sample of seroprevalence (20/50) in cattle from Kasese was estimated in 2017. These results indicate an increase of seroprevalence in livestock from Kasese district and more specifically in locations re-sampled after 2016, which is also consistent with lower percent inhibition in the cELISA (Table 2; Figure 2; Figure 4).

**Figure 5:**
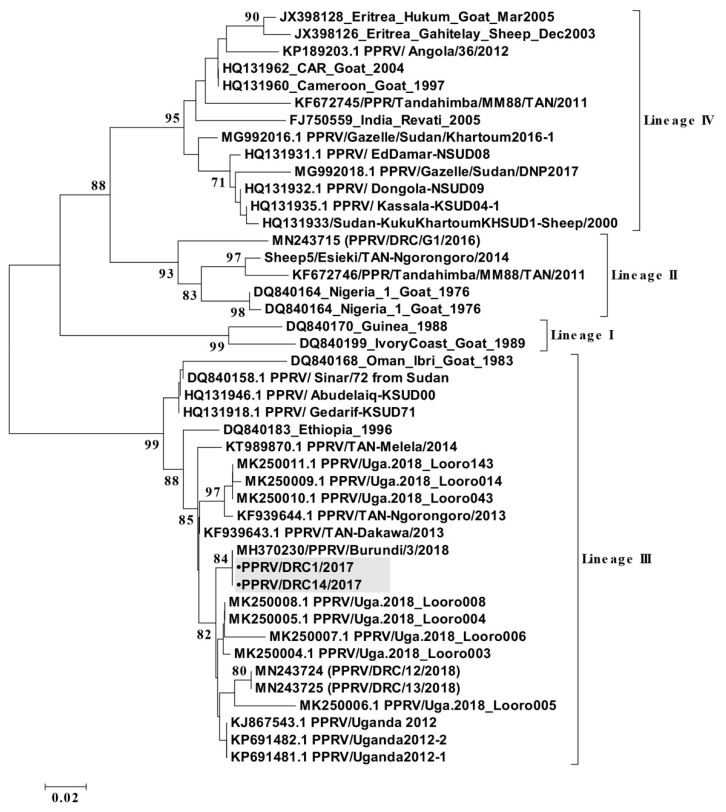
A neighbor-joining tree constructed using partial N-gene (255 nucleotides) sequences of peste des petits ruminants virus (PPRV), shows the relationships among the PPRV isolates. The Kimura 2-parameter model was used to calculate the percentage (indicated by numbers beside branches) of replicate trees in which the associated taxa clustered together in 1000 bootstrap replicates. The sequences from DRC generated in this study PPRV/DRC/01/2017 (MT154039) and PPRV/DRC/14/2017 (MT154040) are indicated with a shaded grey box, were identical to the Burundi sequence (MH370230) and related to other isolates from north-eastern Uganda and eastern DRC 2018 (MN2434724-25). The scale bar indicates nucleotide substitutions per site.

PPR was clinically reported during the study period in small livestock from Uganda in Kasese district (spring; 2016), Kabale and Kisoro districts (June–August; 2017), and North Kivu (October; 2017) in DRC (Figure 1; Figure S1). From the outbreak in North Kivu, 12 nasal swab samples out of 14 tested were found positive in RT-qPCR assay with cycle-threshold values (C_T_-values) ranging from 18 to 34 (Table 3). Of these, four samples with low C_T_-values were subjected to RT-PCR to amplify the C-terminal region of the N-gene. The purified amplicon was sequenced on both strands, and a total of two partial N-gene sequences were generated in this study. A phylogenetic analysis was carried out using the total of 35 partial N-gene sequences (Figure 5) that confirmed the circulation of lineage III PPRV in DRC, as also reported in samples collected from domestic goats in 2018 [49]. This partial N-gene sequence generated in this study is 100% identical to the sequence from Burundi (MH370230) [50], whereas four nucleotide differences were observed between this sequence and the sequences from the 2018 PPRV outbreak in DRC (MN243724 and MN243725) [49]. However, this sequence, along with the sequences from DRC collected during the 2018 PPR outbreaks and Uganda in 2012 and 2018 PPR outbreaks form one cluster (Figure 5).

## 4. Discussion

In the recent past, PPRV has regularly caused disease outbreaks in small ruminants within East Africa associated with different virus strains in circulation [1]. The role of wildlife and wildlife–livestock interfaces in PPR epidemiology is still largely unknown in Africa and this gap could be an issue for the PPR global eradication program. In the present study, we describe the occurrence of several PPR-like syndromes and confirm outbreaks in livestock and a broad and recent exposure of wildlife to PPRV in the northern Albertine Rift and Nile basin. Based on local changes of seroprevalence and viral detection, our results indicate that PPRV circulated during the study period and in previous years within a wide geographic and ecologic range in western Uganda, eastern DRC, and South Sudan, involving livestock and exposed wildlife, and including areas with sedentary livestock systems and forested habitats that are not typically associated with PPR in East Africa. This is contemporary information to recent publications on a similar status in Tanzania where widespread infection in small livestock and wildlife was reported in the Ngorongoro Conservation Area [14].

PPRV lineage II, III, and IV have circulated in different countries and regions from East Africa [14,23,42,51]. This study, together with recent works [49,50], reports the circulation of PPRV lineage III in the northern Albertine Rift area causing the first-known PPR outbreaks in this region from 2016 onwards. Our results confirm that PPRV strains from DRC in October 2017 were identical to the lineage III strains that emerged subsequently in Burundi in December 2017 [50]. These results further indicate that national borders are highly porous for disease spread and that PPR outbreaks in East Africa can easily occur on a transboundary basis [51,52]. This transboundary spread is most likely triggered by formal and/or informal movements of small ruminants. However, the role of wildlife in spreading and maintaining PPR or bridging distant livestock populations cannot be ruled out as transboundary protected areas occur between all states in this region (Figure 1). The reported strains from the DRC in 2017 also cluster with strains detected later in 2018 from different locations in North Kivu, suggesting a local PPRV circulation in this sedentary agro-pastoral system [49]. These findings align with recent observations in the Karamoja region in northeastern Uganda; despite being large pastoral systems, separate foci for transmission and PPRV maintenance were identified within relatively small but probably transboundary areas [23]. Our results also support the importance of regionally coordinated actions and locally adapted strategies to stablish effective PPR surveillance and control.

Sero-epidemiological data from western Uganda indicate similar temporal patterns of PPRV exposure in wildlife and livestock. The first evidence of PPRV infection in wildlife in the area, but without clinical disease, was reported in African buffalo from 2004–2005 (Table S2) [53]. This was also when PPR was first reported in sheep and goats in Soroti, Uganda [19]; while official confirmation of the disease in Uganda had to wait until 2007 (OIE 2007). In this study, we further confirm PPRV antibodies in wildlife from QENP in 2015 with seropositive <1-year-old buffalo and kobs, and subsequent positive livestock in communities around QENP in 2016. The local rise of seroprevalence detected in livestock from 2016 is consistent with a previous study [54], and with PPR compatible clinical syndromes (running nose, depression) and high mortalities of sheep and goats described by local people in Kasese district in spring 2016 (Figure 1; Table S1). These results suggest that wildlife from QENP was widely exposed to PPRV before the first epizootic spread in most of the local livestock, but with a tight temporal pattern. Previous studies in Tanzania also found a linkage between PPRV antibodies in wildlife and epidemic cycles in domestic sheep and goats, suggesting that spillover may easily occur at the wildlife–livestock interface in the African context [14]. Unfortunately, and despite evidences regarding a wildlife–livestock epidemiological link, our study cannot confirm inter-species spillover and the direction of it. 

No evidence of PPRV circulation was detected in the southwest Kisoro district of Uganda by June 2017, and the few seropositive animals detected may be explained by animals moved from other regions. These serum samples predated the first recorded PPR outbreak in Kisoro-Kabale districts in later June through August 2017. This outbreak in Uganda was likely related to the subsequent outbreak in the adjacent North Kivu province of DRC about two months later in 2017 (Figure 1). These areas of western Uganda and northeastern DRC have both road and natural connectivity through the Greater Virunga Landscape.

PPRV antibodies in buffalo and kobs from QENP did not show a clear spatial pattern. If African buffalo are relatively sedentary and Uganda kob at times territorial, these animals in QENP can be considered as one population whereas they may represent at least two epidemiological units divided by the extensive Maramagambo Forest (Figure 2). Buffalo, due to their preference for burned pasture and need for wallows [55], may increase the opportunity for contact with cattle brought into or adjacent to the park [33]. Kob are frequently poached, and are less frequently seen in proximity to livestock when compared to buffalo [33]. Surprisingly in Uganda, PPR infection occurred in all ecologically discrete wildlife populations sampled in both segments of the protected area (north and south Maramagambo Forest and Kazinga Channel; Figure 2). This suggests a consistent force of infection in all areas even though livestock are not allowed to enter the park, and the low intensity of the wildlife–small livestock interface observed. Therefore, at least a temporary PPRV transmission within natural areas, and possibly involving a multi-host system, is likely. The age prevalence data from wildlife are suggestive of discrete events for PPRV transmission within parks, perhaps predictable alongside epidemic cycles in livestock. However, it does not support a significant degree of temporal persistence of PPRV within natural systems. Alternatively, a separate source of morbillivirus exposure may also exist, cross reacting in the N ELISA test (60% of N-gene is conserved between morbilliviruses): this could be the canine distemper virus or another yet unknown morbillivirus. Our results are somewhat different to previous studies that indicated an increased seroprevalence by age in wildlife from Ngorongoro Conservation Area, together with a continued PPRV detection in local domestic sheep and goats [14,56]. Despite all these evidences, the absence of detection of viral RNA in these samplings precludes molecular epidemiology and it remains inconclusive as to the circulation of the virus amongst these wildlife populations.

In South Sudan, some of the wildlife populations, particularly antelope and common warthog (*Phacochoerus africanus*), are highly spatially integrated with livestock even if direct physical contact is rare. Livestock is predominantly nomadic, with large seasonal movements including large flocks of small livestock at times and moving transboundary. These pastoral systems are extensive and show ample opportunity for spillover with frequent livestock infection cycles (Table S1). Adult tiang had a higher sample prevalence than the rest of the wildlife tested from South Sudan, and this may be explained by an increased likelihood of being exposed to PPRV because of its migratory behavior [57]. The Government of South Sudan reported clinical evidence of PPR outbreaks in sheep and goats between 2005 and 2012 in areas surrounding the tiang migration corridor (Adwok personal communication, MARF South Sudan 2014; Table S1). Migratory and long movement patterns may predispose some species to a higher PPR-exposure risk and/or bridge between distant susceptible populations.

Cattle are more frequent visitors to QENP and more likely to share resources and contact with wildlife [33]. Sheep and goats typically dwell nearby human settlements and rarely enter protected areas. It is noteworthy that the prevalence of anti-PPRV antibodies in livestock around QENP is consistent with a recent study based on the same Ugandan districts, and that a higher seroprevalence in cattle as compared to sheep and goats was also found [54]. In northern Tanzania, cattle from villages where PPR occurred among small ruminants also showed high seropositivity [56]. Experimental infection studies suggest cattle are infected sub-clinically [11,58] and this is supported by field data in this study and previous ones [54,59]. However, the competence of cattle to transmit PPRV to other susceptible hosts is poorly explored, especially considering that breeds in livestock may introduce variability in PPR expression and epidemiology [60]. Cattle may be a good sentinel for PPRV circulation at the wildlife–livestock interface given that the infection does not cause fatalities and they are more commonly in contact with wildlife.

The absences of both virus isolation and observation of clinical disease in wildlife species in Africa is surprising given the increasingly visible epidemics in wildlife in Asia [5,6,61,62]. Both absences could reflect facts (absence of viral excretion and disease expression in African wildlife) but could also be due to imperfect datasets constrained by the difficulty to survey disease in wildlife. Mortalities seen in phylogenetically closely related species to the abundant wildlife in East Africa (e.g., saiga antelope (*Saiga tatarica mongolica*)) are not dissimilar to that experienced with RP in wildlife, historically with high morbidity and mortality, in the case of saiga approaching 100% and 80%, respectively [5]. The closest African relatives of the saiga antelope, Thomson’s gazelle (*Eudorcas thomsoni*), are well represented in Kenya and Tanzania, and no sign of disease in PPRV prevalent areas was seen or reported by wildlife or veterinary authorities, or even anecdotally by local communities. This apparent absence of disease in most populations of free-ranging African wildlife is consistent across all species shown to be exposed to PPRV by this study or previous serological studies [14], whilst PPR syndromes in captive African species in the Middle Eastern collections are reported [27]. A recent outbreak of PPR in dorcas gazelle in Dinder National Park, Sudan is the first verifiable report of disease in free-ranging antelope in sub-Saharan Africa, but more pathological and epidemiological data are needed to confirm the laboratory findings [32]. A shift in PPR epidemiology as wildlife becomes increasingly stressed by rising human and livestock numbers and associated loss of forage and disturbance, could be a concerning development for wildlife economies in East Africa that need to be vigilant [4,32]. This is certainly the characteristic of the Asian ecosystems where PPR is readily expressed in wildlife. To explain this phenomenon, more research on the pathogenesis of PPR in wildlife species is needed; in addition, the role of infective strains, nutrition, stress, co-infections, environmental factors, and other ecological aspects need to be better explored to understand the PPR disease ecology. While there is a possibility that wildlife can maintain and bridge viruses between populations of wildlife and livestock [63], attempts at elimination of PPRV might be frustrated. Both livestock and wildlife components should be considered in further field studies to clarify PPR epidemiology, and eco-region level approaches should be implemented to better understand PPR dynamics at a relevant landscape level.

## Figures and Tables

**Table 1:** Results of peste des petits ruminants virus (PPRV) antibody detection in sera from apparently healthy wild ungulates captured in Queen Elizabeth National Park, Uganda in January 2015 and October 2017, and in Nile basin and Boma National Park, South Sudan in 2013.

	Scientific Name	Year	Individuals			Herds		
			*n*	Positives	Prev.% (CI 95%)	*n*	Positives	Prev.% (CI 95%)
**Uganda—Queen Elizabeth National Park**								
Buffalo	*Syncerus caffer*	2015	28	6	21.4 (10.2–39.5)	14	4	28.6 (11.7–54.6)
		2017	30	5	16.7 (7.3–33.6)	14	5	35.7 (16.3–61.2)
		Total	58	11	19.0 (10.9–30.8)	28	9	32.1 (17.9–50.7)
Uganda kob	*Kobus kob thomasi*	2015	39	4	10.3 (4.1–23.6)	20	3	15.0 (5.2–36.0)
All species			97	15	15.5 (9.6–24.0)	48	13	27.1 (16.6–41.0)
**South Sudan—Nile basin and Boma National Park**								
Eland	*Taurotragus oryx*	2013	9	0	0.0 (0.0–29.9)	NA	NA	NA
Elephant	*Loxodonta africana*	2013	40	1	2.5 (0.1–12.9)	NA	NA	NA
Tiang	*Damaliscus lunatus tiang*	2013	21	15	71.4 (50.0–86.2)	NA	NA	NA
White eared Kob	*Kobus kob leucotis*	2013	17	0	0.0 (0.0–18.4)	NA	NA	NA
All species			87	16	18.4 (11.6–27.8)			

*n* = total of animals sampled; Prev. = prevalence; CI = confidence interval.

**Table 2:** Results of PPRV antibody detection in livestock from communities around natural protected areas in western Uganda (MFNP = Murchison Falls National Park, QENP = Queen Elizabeth National Park, MGNP = Mgahinga Gorilla National Park). The sample prevalence is estimated using specificity and sensitivity of the diagnostic test for domestic species [39].

		2015 January–February		2016 June–July		2017 June–August		Total
	*n*	Prev.% (CI 95%)	*n*	Prev.% (CI 95%)	*n*	Prev.% (CI 95%)	*n*	Prev.% (CI 95%)
**Goats**								
MFNP—Bulisa district	15	0.0 (0.0–20.7)	NA	NA	NA	NA	15	0.0 (0.0–20.7)
QENP—Kasese district	15	0.0 (0.0–20.7)	23	0.0 (0.0–14.2)	160	9.6 (5.6–15.6)	198	7.6 (4.3–12.5)
QENP—Rubirizi district	72	0.0 (0.0–4.3)	20	4.2 (0.0–24.2)	0	NA	92	0.1 (0.0–5.2)
MGNP—Kisoro district	59	0.0 (0.0–5.5)	52	1.0 (0.0–9.7)	139	0.0 (0.0–1.8)	250	0.0 (0.0–1.3)
**Sheep**								
MFNP—Bulisa district	5	0.0 (0.0–45.4)	0	NA	0	NA	5	0.0 (0.0–45.4)
QENP—Kasese district	0	NA	7	29.5 (7.7–67.5)	20	20.3 (7.6–43.4)	27	22.2 (10.3–42.5)
QENP—Rubirizi district	8	0.0 (0.0–33.6)	6	0.0 (0.0–40.7)	0	NA	14	0.0 (0.0–22.0)
MGNP—Kisoro district	53	0.0 (0.0–6.2)	47	3.5 (0.2–14.2)	107	0.0 (0.0–4.4)	207	0.5 (0.0–3.4)
**Cattle**								
MFNP—Bulisa district	0	NA	0	NA	0	NA	0	NA
QENP—Kasese district	137	2.8 (0.6–7.8)	60	39.9 (27.9–53.5)	50	39.6 (26.6–54.4)	247	19.3 (14.6–25.0)
QENP—Rubirizi district	3	0.0 (0.0–60.0)	0	NA	0	NA	3	0.0 (0.0–60.0)
MGNP—Kisoro district	39	0.0 (0.0–8.5)	51	3.1 (0.1–13.1)	146	0.0 (0.0–3.0)	236	0.3 (0.0–2.9)

*n* = total of animals sampled; Prev. = prevalence; CI = confidence intervalv.

**Table 3:** Information of the nasal swab samples collected on 20 October 2017 from small domestic ruminants in Democratic Republic of the Congo and respective C_T_-values (cycle-threshold values) obtained in the RT-qPCR assay.

Sample No.	Species	Sex	Age	C_T_-Value
DRC-1	Goat	F	10 months	18.16
DRC-2	Goat	F	4 years	29.10
DRC-3	Goat	F	4 years	32.79
DRC-4	Goat	F	3 years	28.06
DRC-5	Goat	F	1.5 years	24.79
DRC-6	Goat	F	1 year	34.11
DRC-7	Goat	F	1 year	29.68
DRC-8	Sheep	F	2 years	UD
DRC-9	Sheep	F	2 years	32.11
DRC-10	Sheep	F	3 years	25.09
DRC-11	Sheep	M	1.5 years	UD
DRC-12	Sheep	F	4 years	27.67
DRC-13	Goat	F	3 years	22.17
DRC-14	Goat	F	1 year	20.23

F = female; M = male; UD = not detected.

## Supplementary Materials

Supplementary materials can be found at https://www.mdpi.com/1999-4915/12/3/293/s1. History of PPR in East Africa, characteristics of natural and livestock systems in study areas from Uganda, South Sudan, and Democratic Republic of the Congo, Table S1: Historical records on peste des petits ruminants outbreaks and evidence of viral exposure in Uganda, South Sudan, and Democratic Republic of the Congo, Table S2: Retrospective information on peste des petits ruminant antibody detection in wild artiodactyls from naturally protected areas in Uganda under the Pan African Programme for the Control of Epizootics (PACE), Table S3: Drugs used for wildlife captures in Uganda (buffalo and Uganda kob) and South Sudan (tiang, white eared kob, eland, and elephant), Table S4: Number of herd owners sampled in western Uganda, Figure S1: Clinical signs observed in the outbreak of disease with a PPR-like syndrome in Kabale and Kisoro districts from western Uganda in 2017.

## References

[1] Parida S., Muniraju M., Mahapatra M., Muthuchelvan D., Buczkowski H., Banyard A.C. (2015). Peste des petits ruminants. Vet. Microbiol..

[2] OIE-FAO (2015). Global Control and Eradication of Peste des Petits Ruminants. Investing in Veterinary Systems, Food Security and Poverty Alleviation. http://www.fao.org/3/a-i4477e.pdf.

[3] Jones B.A., Rich K.M., Mariner J.C., Anderson J., Jeggo M., Thevasagayam S., Cai Y., Peters A.R., Roeder P. (2016). The economic impact of eradicating peste des petits ruminants: A benefit-cost analysis. PLoS ONE.

[4] Aguilar X.F., Fine A.E., Pruvot M., Njeumi F., Walzer C., Kock R., Shiilegdamba E. (2018). PPR virus threatens wildlife conservation. Science.

[5] Pruvot M., Strindberg S., Shiilegdamba E., Ganzorig K., Damdinjav B., Buuveibaatar B., Chimeddorj B., Bayandonoi G., Jargalsaikhan T., Shatar M. (2020). Outbreak of Peste des Petits Ruminants among Critically Endangered Mongolian Saiga and Other Wild Ungulates, Mongolia, 2016–2017. Emerg. Infect. Dis..

[6] Kock R., Crisis Management Centre for Animal Health Mission Report (2017). Mongolia. Investigation of Peste des Petits Ruminants (PPR) among Wild Animals and Its Potential Impact on the Current PPR Situation in Livestock.

[7] Parida S., Muniraju M., Altan E., Baazizi R., Raj G.D., Mahapatra M. (2016). Emergence of PPR and its threat to Europe. Small Rumin. Res..

[8] Pope R.A., Parida S., Bailey D., Brownlie J., Barrett T., Banyard A.C. (2013). Early Events following Experimental Infection with Peste-Des-Petits Ruminants Virus Suggest Immune Cell Targeting. PLoS ONE.

[9] Truong T., Boshra H., Embury-Hyatt C., Nfon C., Gerdts V., Tikoo S., Babiuk L.A., Kara P., Chetty T., Mather A. (2014). Peste des petits ruminants virus tissue tropism and pathogenesis in sheep and goats following experimental infection. PLoS ONE.

[10] Parida S., Couacy-Hymann E., Pope R.A., Mahapatra M., El Harrak M., Brownlie J., Banyard A.C., Munir M. (2015). Pathology of Peste des Petits Ruminants. Peste Des Petits Ruminants Virus.

[11] World Organization for Animal Health (OIE) (2013). Peste des petits ruminants (infection with peste des petits ruminants virus). Manual of Diagnostic Tests and Vaccines for Terrestrial Animals 2019.

[12] Banyard A.C., Wang Z., Parida S. (2014). Peste des petits ruminants virus, Eastern Asia. Emerg. Infect. Dis..

[13] OIE-FAO (2015) Global Strategy for the Control and Eradication of PPR, World Organization for Animal Health, United Nations Food and Agriculture Organisation. http://www.oie.int/eng/ppr2015/doc/PPR-Global-Strategy-2015-03-28.pdf.

[14] Mahapatra M., Sayalel K., Muniraju M., Eblate E., Fyumagwa R., Shilinde L., Mdaki M., Keyyu J., Parida S., Kock R. (2015). Spillover of Peste des Petits Ruminants Virus from Domestic to Wild Ruminants in the Serengeti Ecosystem, Tanzania. Emerg. Infect. Dis..

[15] El Hag Ali B., Taylor W. (1984). Isolation of peste des petits ruminants virus from the Sudan. Res. Vet. Sci..

[16] Roeder P.L., Abraham G., Kenfe G., Barretf T. (1994). Peste des Petits Ruminants in Ethiopian Goats. Trop. Anim. Health Prod..

[17] Wamwayi H.M., Rossiter P.B., Kariuki D.P., Wafula J.S., Barrett T., Anderson J. (1995). Peste des petits ruminants antibodies in East Africa. Vet. Rec..

[18] Karimuribo E.D., Loomu P.M., Mellau L.S.B., Swai E.S. (2011). Retrospective study on sero-epidemiology of peste des petits ruminants before its official confirmation in northern Tanzania in 2008. Res. Opin. Anim. Vet. Sci..

[19] (2004). AU-IBAR Pan African Animal Health Yearbook 2003.

[20] Luka P.D., Erume J., Mwiine F.N., Ayebazibwe C. (2012). Molecular characterization of peste des petits ruminants virus from the Karamoja region of Uganda (2007–2008). Arch. Virol..

[21] Banyard A.C., Parida S., Batten C., Oura C., Kwiatek O., Libeau G. (2010). Global distribution of peste des petits ruminants virus and prospects for improved diagnosis and control. J. Gen. Virol..

[22] Torsson E., Kgotlele T., Berg M., Mtui-Malamsha N., Swai E.S., Wensman J.J., Misinzo G. (2016). History and current status of peste des petits ruminants virus in Tanzania. Infect. Ecol. Epidemiol..

[23] Nkamwesiga J., Coffin-Schmitt J., Ochwo S., Mwiine F.N., Palopoli A., Ndekezi C., Isingoma E., Nantima N., Nsamba P., Adiba R. (2019). Identification of peste des petits ruminants transmission hotspots in the Karamoja subregion of Uganda for targeting of eradication interventions. Front. Vet. Sci..

[24] Dundon W.G., Kihu S.M., Gitao G.C., Bebora L.C., John N.M., Oyugi J.O., Loitsch A., Diallo A. (2015). Detection and Genome Analysis of a Lineage III Peste Des Petits Ruminants Virus in Kenya in 2011. Transbound. Emerg. Dis..

[25] Kihu S.M., Gachohi J.M., Ndungu E.K., Gitao G.C., Bebora L.C., John N.M., Wairire G.G., Maingi N., Wahome R.G., Ireri R. (2015). Sero-epidemiology of Peste des petits ruminants virus infection in Turkana County, Kenya. BMC Vet. Res..

[26] Munir M. (2014). Role of Wild Small Ruminants in the Epidemiology of Peste Des Petits Ruminants. Transbound. Emerg. Dis..

[27] Wensman J.J., Abubakar M., Shabbir M.Z., Rossiter P. (2018). Peste des petits ruminants in wild ungulates. Trop. Anim. Health Prod..

[28] Li J., Li L., Wu X., Liu F., Zou Y., Wang Q., Liu C., Bao J., Wang W., Ma W. (2017). Diagnosis of Peste des Petits Ruminants in Wild and Domestic Animals in Xinjiang, China, 2013–2016. Transbound. Emerg. Dis..

[29] Marashi M., Masoudi S., Moghadam M.K., Modirrousta H., Marashi M., Parvizifar M., Dargi M., Saljooghian M., Homan F., Hoffmann B. (2017). Peste des petits ruminants virus in vulnerable wild small ruminants, Iran, 2014–2016. Emerg. Infect. Dis..

[30] Couacy-Hymann E., Bodjo C., Danho T., Libeau G., Diallo A. (2005). Surveillance of wildlife as a tool for monitoring rinderpest and peste des petits ruminants in West Africa. Rev. Sci. Tech..

[31] Kock R.A., Wamwayi H.M., Rossiter P.B., Libeau G., Wambwa E., Okori J., Shiferaw F.S., Mlengeya T.D. (2006). Re-infection of wildlife populations with rinderpest virus on the periphery of the Somali ecosystem in East Africa. Prev. Vet. Med..

[32] Asil R.M., Ludlow M., Ballal A., Alsarraj S., Ali W.H., Mohamed B.A., Mutwakil S.M., Osman N.A. (2019). First detection and genetic characterization of peste des petits ruminants virus from dorcas gazelles “*Gazella dorcas*” in the Sudan, 2016-2017. Arch. Virol..

[33] Meunier N.V., Sebulime P., White R.G., Kock R. (2017). Wildlife-livestock interactions and risk areas for cross-species spread of bovine tuberculosis. Onderstepoort J. Vet. Res..

[34] Miguel E., Grosbois V., Caron A., Boulinier T., Fritz H., Cornelis D., Foggin C., Makaya P.V., Tshabalala P.T., De Garine-Wichatitsky M. (2013). Contacts and foot and mouth disease transmission from wild to domestic bovines in Africa. Ecosphere.

[35] Fenton A., Pedersen A.B. (2005). Community epidemiology framework for classifying disease threats. Emerg. Infect. Dis..

[36] Plumptre A.J., Davenport T.R.B., Behangana M., Kityo R., Eilu G., Ssegawa P., Ewango C., Meirte D., Kahindo C., Herremans M. (2007). The biodiversity of the Albertine Rift. Biol. Conserv..

[37] Olupot W., Mcneilage A.J., Plumptre A.J. (2009). An Analysis of Socioeconomics of Bushmeat Hunting at Major Hunting Sites in Uganda.

[38] Kock R.A., Barrett T., Pastoret P.-P., Taylor W. (2005). Rinderpest and wildlife. Rinderpest and Peste des Petits Ruminants Virus. Plagues of large and small ruminants.

[39] Libeau G., Lancelot R., Colas F., Guerre L., Bishop D.H.L., Diallo A. (1995). Development of competitive ELISA for the peste des petits ruminants virus using a recombinant nucleoprotein. Res. Vet. Sci..

[40] Clarke B., Mahapatra M., Friedgut O., Bumbarov V., Parida S. (2017). Persistence of Lineage IV Peste-des-petits ruminants virus within Israel since 1993: An evolutionary perspective. PLoS ONE.

[41] Batten C.A., Banyard A.C., King D.P., Henstock M.R., Edwards E., Sanders A., Buczkowski H., Chris Oura C.L., Barrett T. (2011). A real time RT-PCR assay for the specific detection of Peste des petits ruminants virus. J. Virol. Methods.

[42] Muniraju M., Munir M., Parthiban A.R., Banyard A.C., Bao J., Wang Z., Ayebazibwe C., Ayelet G., El Harrak M., Mahapatra M. (2014). Molecular Evolution of Peste des Petits Ruminants Virus. Emerg. Infect. Dis..

[43] Baazizi R., Mahapatra M., Clarke B.D., Ait-Oudhia K., Khelef D., Parida S. (2017). Peste des petits ruminants (PPR): A neglected tropical disease in maghreb region of North Africa and its threat to Europe. PLoS ONE.

[44] Kimura M. (1980). A simple method for estimating evolutionary rates of base substitutions through comparative studies of nucleotide sequences. J. Mol. Evol..

[45] Tamura K., Stecher G., Peterson D., Filipski A., Kumar S. (2013). MEGA6: Molecular evolutionary genetics analysis version 6.0. Mol. Biol. Evol..

[46] Stevenson M., Nunes T., Heuer C., Marshall J., Sanchez J., Thornton R., Reiczigel J., Robison-Cox J., Sebastiani P., Solymos P. Package epiR: An R Package for the Analysis of Epidemiological Data. R package version 1.0-11. https://cran.r-project.org/web/packages/epiR/epiR.pdf.

[47] R Development Core Team (2017). R: A Language and Environment for Statistical Computing.

[48] QGIS Development Team QGIS 2016. Las Palmas 2.18. Geogaphic Information System. Open Source Geospatial Foundation Project. http://qgis.osgeo.org.

[49] Tshilenge M.G., Walandila J.S., Byakya D., Masumu J., Katshay L., Cattoli G., Bushu E., Mpiana S., Dundon W.G. (2019). Peste des petits ruminants viruses of lineages II and III identified in the Democratic Republic of the Congo. Vet. Microbiol..

[50] Niyokwishimira A., de D Baziki J., Dundon W.G., Nwankpa N., Njoroge C., Boussini H., Wamwayi H., Jaw B., Cattoli G., Nkundwanayo C. (2019). Detection and molecular characterization of Peste des Petits Ruminants virus from outbreaks in Burundi, December 2017–January 2018. Transbound. Emerg. Dis..

[51] Misinzo G., Kgotlele T., Muse E.A., Van Doorsselaere J., Berg M., Munir M. (2015). Peste des Petits Ruminants Virus Lineage II and IV from Goats in Southern Tanzania During an Outbreak in 2011. Br. J. Virol..

[52] Tounkara K., Kwiatek O., Niang M., Abou Kounta Sidibe C., Sery A., Dakouo M., Salami H., Lo M.M., Ba A., Diop M. (2019). Genetic Evidence for Transboundary Circulation of Peste Des Petits Ruminants Across West Africa. Front. Vet. Sci..

[53] Kock R.A. (2008). The Role of Wildlife in the Epidemiology of Rinderpest in East and Central Africa 1994-2004: A Study Based on Serological Surveillance and Disease Investigation. Ph.D. Thesis.

[54] Nakayima J., Nanfuka M., Kidega E., Kajuna Y., Ndumu D. (2018). Epidemiological Insights into the Occurrence of Peste des Petits Ruminants Virus (PPRV) Among Sheep, Goats and Cattle in Western Uganda. Int. J. Livest. Res..

[55] Field C.R., Laws R.M. (1970). The distribution of the larger herbivores in the Queen Elizabeth National Park, Uganda. J. Appl. Ecol..

[56] Lembo T., Oura C., Parida S., Hoare R., Frost L., Fyumagwa R., Kivaria F., Chubwa C., Kock R., Cleaveland S. (2013). Peste des petits ruminants infection among cattle and wildlife in northern Tanzania. Emerg. Infect. Dis..

[57] Marjan M.D. (September 2014). Movements and Conservation of the Migratory white-eared kob (*Kobus kob leucotis*) in South Sudan. Ph.D. Thesis.

[58] Sen A., Saravanan P., Balamurugan V., Bhanuprakash V., Venkatesan G., Sarkar J., Rajak K.K., Ahuja A., Yadav V., Sudhakar S.B. (2014). Detection of subclinical peste des petits ruminants virus infection in experimental cattle. Virus Dis..

[59] Agga G.E., Raboisson D., Walch L., Alemayehu F. (2019). Epidemiological Survey of Peste des Petits Ruminants in Ethiopia: Cattle as Potential Sentinel for Surveillance. Front. Vet. Sci..

[60] Fakri F.Z., Elhajjam A., Bamouh Z., Jazouli M., Boumart Z., Tadlaoui K., Fassi-Fihri O., Elharrak M. (2017). Susceptibility of Moroccan sheep and goat breeds to peste des petits ruminants virus. Acta Vet. Scand..

[61] Hoffmann B., Wiesner H., Maltzan J., Mustefa R., Eschbaumer M., Arif F.A., Beer M. (2011). Fatalities in Wild Goats in Kurdistan Associated with Peste Des Petits Ruminants Virus. Transbound. Emerg. Dis..

[62] Abubakar M., Rajput Z.I., Arshed M.J., Sarwar G., Ali Q. (2011). Evidence of peste des petits ruminants virus (PPRV) infection in Sindh Ibex (*Capra aegagrus blythi*) in Pakistan as confirmed by detection of antigen and antibody. Trop. Anim. Health Prod..

[63] Caron A., Cappelle J., Cumming G.S., De Garine-Wichatitsky M., Gaidet N. (2015). Bridge hosts, a missing link for disease ecology in multi-host systems. Vet. Res..

