# Supplementary material for: Peste des Petits Ruminants at the Wildlife–Livestock Interface in the Northern Albertine Rift and Nile Basin, East Africa
Source: Viruses. 2020 Mar 7;12(3):293. doi: 10.3390/v12030293 (PMC7150925; doi:10.3390/v12030293)
Supplement: Supplementary file 1 [file viruses-12-00293-s001.pdf]

Supplementary Material - Peste des Petits Ruminants at the Wildlife-Livestock Interface in the Northern Albertine Rift and Nile Basin, East Africa

History of PPR in East Africa

The first PPR cases described in livestock from East Africa date from 1971-1972 in Sudan [1], followed by outbreaks in Ethiopia, suspected since 1977 and confirmed in 1989-1990 [2]. Serology also indicated the exposure of sheep and goats to PPRV from the Karamoja region of northeastern Uganda in 1985 and in northern and western regions of Kenya in 1987-1991 [3], suggesting that PPRV may have had occasional incursion into other East African countries. An endemic situation with almost yearly incidence of PPR livestock outbreaks was already established by 2000 in Sudan and Ethiopia [4,5], but based on serology of wild animal populations, the virus apparently did not persist further south in East Africa (Table S2) [6]. This apparent barrier may well reflect the continued circulation of RPV in East Africa up until 2002 and vaccination beyond until its final elimination reported in 2011 [7]. Rinderpest is a close relative to PPR, serologically indistinguishable by some ELISAs and for which infection is cross protective.

The first PPR cases described this century in Uganda were in Soroti district in 2003, without much epidemiological information [8]. This corresponds with previous reports of PPR cases in areas from South Sudan (former Sudan) near to the Ugandan border in 2002 and suspected PPR and antibody detection in livestock from Tanzania 2004 (Table S1) [9]. In 2006-2008, the first official and large PPR outbreak was reported in Kenya and was also associated with disease in the bordering Uganda region of Karamoja in 2007-2008 and northern Tanzania in 2008 [4,9,10]. Uganda reported more than 180,000 disease cases and about 7,200 losses in that outbreak, numbers that are likely underestimated [11,12]. Further outbreaks in Uganda occurred in northern districts of Lamwo and Abim in 2010 with about 3,800 losses [13] and other locations throughout 2011-2016 but with limited epidemiological information [14–18]. PPR appeared to be absent in Ugandan wildlife prior to 2004 from serosurveillance under the PARC/PACE programs for RP eradication, but African buffalo (*Syncerus caffer*) turned seropositive in analyses of convenience samples from 2004-2005 in Kidepo (8% prevalence), Murchison Falls National Park (MFNP, 24% prevalence) and Queen Elizabeth National Park (QENP, 25% prevalence), corresponding with the first reports of PPR in livestock (Table S1, Table S2). All the evidence suggests that PPR was probably present in some parts of the country and causing outbreaks of disease when introduced to naïve areas since about 2004. This scenario was supported by serosurveys in 2009, 2011 and 2016 that provided a heterogeneous image with PPRV antibodies in small livestock from most of the northern and eastern Uganda districts, and a wider spread

of the virus than previously assumed throughout the country [13,18,19]. A similar widespread but spatially discrete pattern of PPRV antibodies is described in national surveys of Ethiopia [20] and Tanzania [21]. The consistent detection of PPRV or PPRV-antibodies in some nomadic and semi-nomadic pastoralist systems from South Sudan, north-eastern Uganda, north-western Kenya and northern Tanzania suggests that the virus may persist locally or by encompassing larger transboundary areas (Table S1) [21–24].

Characteristics of natural and livestock systems in study areas from Uganda, South Sudan and Democratic Republic of the Congo

Western Uganda

Queen Elizabeth Conservation Area and Murchison Falls Conservation Area include their respective national parks and are the largest savanna ecosystems in western Uganda with an area of 2,401 km^2 and 5,045 km^2 respectively [25]. Mgahinga National Park (MGNP) is a small volcano area of 33.7 km^2 with high tropical forest located in the southwest corner of Uganda, bordering with Rwanda and DRC (Figure 1).

Buffalo and Uganda kob (*Kobus kob thomasi*) are the predominant wild ungulates in open landscapes from western Uganda and neighboring regions, with an estimated population of 10,721 buffalo and 15,469 Uganda kob in the Queen Elizabeth National Park (QENP) in 2016 [26]. These two gregarious species have local movement patterns within QENP [27], but limited migrations may occur to the Virunga National Park in DRC through the north and south sections of Lake Edward [28]. The ecological barriers of Kazinga channel and Maramagambo forest in QENP apparently restrict wild ungulate movements and contacts from the northern and southern savanna sectors [29] (Figure 2). Other common artiodactyls in QENP are hippopotamus (*Hippopotamus amphibius*), waterbuck (*Kobus ellipsiprymnus*), warthog (*Phacochoerus africanus*) and topi (*Damaliscus lunatus*). Presence of potentially susceptible forest species such as bushbuck (*Tragelaphus scriptus*), duikers (*Cephalophus* spp.) or forest hog (*Hylochoerus meinertzhageni*) is notable and potentially might provide bridge hosts across the forest ecosystems.

Human population in sub-counties around QENP (Kihiihi, Bwambara, Kyamuhunga, Rutoto, Ryeru, Kicwamba, Kirugu, Katunguru, Katerera, Kyabakara, Muhokya, Karusandara, Nyamwamba, Kasese, Kanara, Nyakatonzi, Katwe-Kabatoro, Lake Katwe, Nyakyumbu and Isango) was estimated at 440,862 in 2014, mostly located in the counties adjacent to the northern and western border of the park [30]. The livestock system of western Uganda is markedly different to the pastoral systems of South Sudan, eastern Uganda and Kenya where PPR is more usually associated. Activities typically involve agriculture

and livestock, which is predominantly structured on small household herds for subsistence production [31]. Livestock presence by household around QENP are mainly goats in 64% of the households, followed by cattle in 13% and sheep in 11% [25].

The Basongora are traditional cattle pastoralists that settled in 2006-2007 around the northern border of QENP in Kasese district [32]. A total of 11 fishing villages around Lake George and Edwards also occur in small enclaves within the boundaries of QENP and with a varied range of livestock that numbers only a few hundred or thousands. Other communities around QENP are mainly Bakonzo farmers with fewer small livestock around households. Ankole cattle herds from Basongora may be up to 300 animals and perform daily movements around communal pastures bordering the northern part of QENP and inter-mix at water holes and communal grazing areas [32]. In contrast, sheep and goats free-range around human settlements and do not perform large displacements although undoubtedly small numbers of animals are traded and move in and out of the area frequently. The number of livestock is increasing and thus the grazing pressure around the park, leading some farmers to illegally drive their herds inside QENP looking for better pastures [28]. Large mammals in QENP are also following an increasing population trend in the last decades and as a result, a range of indirect interactions with wildlife occur on the border of QENP and its buffer zones [32].

The latest national livestock census in 2008 provided information at district level and estimated 43326 ± 4533.9 goats, 3884 ± 866.3 sheep and 34801 ± 7898.5 cattle in Buliisa district, 227,518 ± 17937.7 goats, 24890 ± 4097.8 sheep and 97243 ± 41548.7 cattle in Kasese district, 376561 ± 25427.8 goats, 79757 ± 5966.8 sheep and 207184 ± 9398.6 cattle in the former Bushenyi district that included the actual Rubirizi district, and 96815 ± 6595.8 goats, 39554 ± 4295.9 sheep and 28083 ± 3115.3 cattle in Kisoro district [31].

South Sudan - Nile Basin

One of Africa’s largest remaining wilderness areas is situated in South Sudan. Boma National Park covers 20,000 Km^2 and is dominated by a plateau covered by woodlands and wooded savanna but also presents grassland plains and swamps; the Sudd wetland is an extensive floodplain that is fed by the White Nile and hosts three protected areas in the north/north west; the Southern National Park is covered by homogeneous woods [33]. The eastern area of the country, encompassing the Sudd and from Bandingilo NP to Boma NP, hosts some of the last major mammal migrations in the world: white-eared kob (*Kobus kob leucotis*), tiang (*Damaliscus lunatus tiang*), and Mongalla gazelle (*Eudorcas albonotata*) for an estimated total 1.2 million animals move freely within an almost uninterrupted ecosystem [33]. Other common wild

artiodactyl species are Bohor reedbuck (*Redunca redunca*), African buffalo, Nile lechwe (*Kobus megaceros*), Grant's gazelle, Lelwel hartebeest (*Alcelaphus buselaphus lelwel*), and warthog [33].

Small and sparse settlements are distributed along rivers in and around the protected areas, mainly near the northern and eastern border of the country. Principal human activities are subsistence pastoralism, fishing, hunting and agriculture; 989,889 cattle and 149,451 sheep and goats were estimated to live in and around the protected areas in 2007 [33]. During two decades of civil war (1983-2005), the military groups have largely exploited wildlife for rations, and buffaloes, zebras and hartebeest populations have been decimated in Boma NP [33]. Migratory species have survived, although tiang numbers have plummeted (Kock, M. personal communication). A 2005 peace agreement put an end to the civil war, but new fighting broke out in 2013 and are still ongoing. With a human population of 8,260,490 in 2009 [34] and a projection of 13,249,924 in 2020 [35], the achievement of a new situation of peace could paradoxically pose other threats to the conservation of biodiversity. During the 2005-2013 period of stability, human activities began to expand in previously untouched areas [33]. The predominant pastoral lifestyle is likely to expand under stability periods along with livestock populations as communities recover and occupy land currently used by wildlife, increasing risk of epizooties caused by livestock diseases.

Eastern Democratic Republic of the Congo

Virunga National Park is located in the eastern DRC, close to the border with Uganda and Rwanda (Figure 1). Thanks to its wide variety of habitats, like glaciers, forests and savannas, it is considered the most biodiverse area of the Albertine Rift Mountains [36]. Wild artiodactyl species populating the park are substantially comparable to those mentioned above for Uganda, with the latest available counts of the most abundant species reporting 4,584 Uganda Kobs, 630 Topi, and 586 Buffaloes in 2014 [26].

The park is located in the North Kivu province, an historically densely populated region with an estimated population of 6,034,208 in 2015 [37]. However, it is very difficult to estimate its actual population, since the most recent census date back to the 1990s [38]. Furthermore, since the second half of the 1990s, the context of war and political instability of the DRC and its neighbouring countries has generated a profound demographic, socioeconomic and urban reconfiguration [38]. In the eastern provinces of North and South Kivu, boomtowns have developed alongside the already established urban centres, especially around refugee and internally displaced person camps, transforming rural landscapes in new urbanised areas [38].

Large livestock was the most affected by the war: most of the cattle were looted or killed. For this reason, livestock activities shifted to raising of small farm animals (poultry, rabbits, goats, swine and guinea pigs), which can be carried more easily by the owners in case of displacement [37]. In 2014, all livestock species were increasing in North Kivu, but they had not yet reached the pre-conflict numbers: 404,390 goats, 328,670 pigs, 277,100 cattle, and 222,320 sheep were counted in the province [37].

Figure S1. Clinical signs observed in the outbreak of disease with a PPR-like syndrome in Kabale and Kisoro districts from western Uganda in 2017. These included depression and respiratory distress with profuse nasal catarrhal exudate and excessive salivation that crusted over the lips and nostrils (A and B), severe diarrhea (C) and corneal opacity (D).

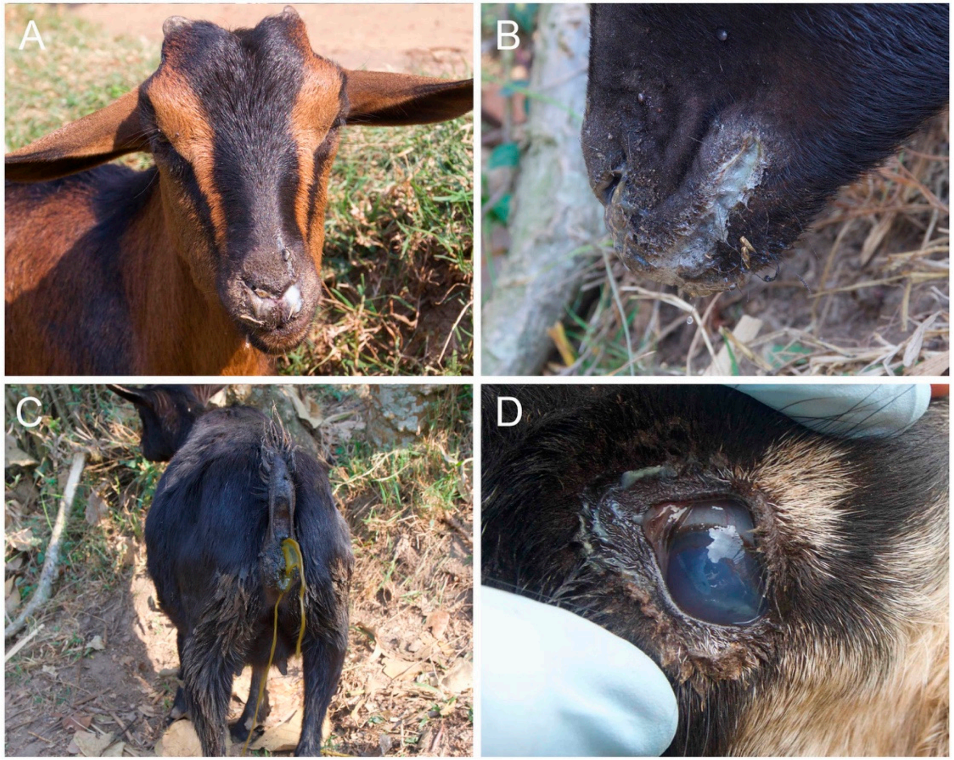

Table S1. Historical records on Peste des petits Ruminants outbreaks and evidence of viral exposure in Uganda, South Sudan and Democratic Republic of the Congo. Numbers on cases and areas affected are as reported or stated in the references but may be unprecise or underestimated. Administrative divisions are indicated as reported and may correspond to former political borders. S: susceptible, C: cases, D: deaths, S/D: slaughtered or destroyed, NA: unknown, VNT: Virus neutralization test; PACE: Pan African Programme for the Control of Epizootics; NADDEC: National Animal Disease Diagnostics and Epidemiology Centre Laboratories (Uganda).

**UGANDA**										
**State or region, district or province, (city)**	**Species**	**Date**	**Outbreaks***	**S**	**C**	**D**	**S/D**	**Lab. confirmation**	**Lineage**	**Reference**
Eastern Region, Karamoja sub-Region	Sheep, goats	1985	Sero-survey	NA	NA	NA	NA	VNT	NA	[3]
Eastern Region, Soroti District	Sheep	2003	1	NA	NA	NA	NA	NA	NA	[8]
Northern and Western Region	African Buffalo	2004-2005	Sero-survey					cELISA VNT		PACE program / Table S1
Northern Region, Karamoja sub-region, Kotido and Kaabong Districts	NA	2006	NA	NA	NA	NA	NA	NA	NA	[39,40]
Northern Region, Karamoja sub-Region, Moroto District	Sheep, goats	03/04/2007	5	NA	8291	2401	4175	cELISA RT-PCR	3	[40,41]
Northern Region, Karamoja sub-Region, Nakapiripirit, Moroto, Kotido, Kaabong and Abim Districts	Sheep, goats	2007	1	NA	NA	NA	NA	RT-PCR	3,4	[10,22]
Northern Region, Karamoja sub-Region, Nakapiripirit, Moroto, Kotido, Kaabong and Abim Districts	Sheep, goats	2008	1	NA	NA	NA	NA	RT-PCR	4	[10,22]
NA	NA	2008	2	NA	177000	617	NA	NA	NA	[12]
Northern Region, Lira, Pader, Kitgum Districts. Eastern Region, Soroti, Katakwi, Kapchorwa, Amuria, Bukedea,	Sheep, goats	2009	Sero-survey					cELISA		[42]

Bukwa, Kaberamaido and Sironko Districts										
Northern Region, Karamoja sub-Region, Moroto, Nakapiripirit, Abim and Kotido Districts	Sheep, goats	2009	Sero-survey					cELISA		[43]
Northern Region, Amuru and Gulu Districts	Sheep, goats	2009	Sero-survey					cELISA		[19]
Northern Region, Abim District	Sheep, goats	09/2010	1	NA	NA	3447	NA	NA	NA	NADDEC
Northern Region, Lamwo District	Goats	05/2010 - 09/2010	1	NA	NA	300	NA	cELISA RT-PCR	NA	NADDEC
Northern Region, 19 districts; Eastern Region, 10 districts; Western Region, 6 districts; Central Region, 1 district. #	Sheep, goats		Sero-survey					cELISA		[13]
NA	NA	2011	4	NA	1375	145	0	NA	NA	[17]
Central Region, Kampala District	Goats	2012						cELISA		[44]
Northern Region, Kotido District.	Goat	2012	NA	NA	NA	NA	NA	RT-PCR	3	[14]
NA	NA	2012	1	NA	NA	NA	NA	RT-PCR	3	[15]
NA	NA	2013	8	83142	559	90	0	NA	NA	[16]
NA	NA	2014	2	1321	773	90	0	NA	NA	[45]
Western Region, Kasese and Rubirizi Districts	Sheep, goats, cattle	2016	Sero-survey					cELISA		[18]
Western Region, Kanungu and Kasese Districts	African Buffalo, Uganda kob	2015, 2017	Sero-survey					cELISA		This study
Western Region, Kasese, Rubirizi and Kisoro Districts	Sheep, goats, cattle	2015 - 2017	Sero-survey					cELISA		This study
Northern Region, Karamoja sub-Region, Loroo Subcounty in Amudat District; Nakapelimoru Subcounty in Kotido District; Kamion Subcounty in Kaabong District	Sheep, goats	2018	NA	NA	NA	NA	NA	cELISA RT-PCR	3	[22]
**SOUTH SUDAN**										

State or region, district or province, (city)	Species	Date	Outbreaks*	S	C	D	S/D	Lab. confirmation	Lineage	Reference
Jonglei State, Akobo county (Nyijule)	Sheep, goats	31/12/2004	NA	NA	NA	NA	NA	No	NA	Veterinary Department
Eastern Equatoria State, Kapoeta East County (Narus)	Goats	10/01/2005	NA	NA	NA	NA	NA	No	NA	Veterinary Department
Jonglei State, Duk County (Poktop)	Sheep, goats	13/02/2005	NA	NA	NA	NA	NA	No	NA	Veterinary Department
Eastern Equatoria State, Budi County (Lotukei)	Goats	25/10/2005	NA	500	127	20	NA	No	NA	Veterinary Department
Eastern Equatoria State, Kapeota East and North Counties	Goats	14/02/2009	NA	1500	60	10	NA	No	NA	Veterinary Department
Northern Bhar Elghazal State, Aweil West County	Sheep	30/01/2010	NA	4000	4	3	NA	No	NA	Veterinary Department
Warab State, Gogrial West County	Goats	01/06/2011	NA	2500	5	1	NA	RT-PCR	4	Veterinary Department
Lakes State, Cueibet county	Sheep, goats	20/04/2012	NA	NA	NA	NA	NA	No	NA	Veterinary Department
Central Equatoria State, Juab County (Kajokij)	Goats	15/06/2012	NA	NA	NA	NA	NA	No	NA	Veterinary Department
Warab State, Gogrial East County	Goats	30/06/2012	NA	NA	NA	NA	NA	No	NA	Veterinary Department
Northern Bhar Elghazal State, Awial East County	Sheep, goats	05/08/2012	NA	NA	NA	NA	NA	No	NA	Veterinary Department
Jonglei State, Twic East County	Goats	25/08/2012	NA	NA	NA	NA	NA	No	NA	Veterinary Department
Warab State, Gogrial West County	Goats	04/09/2012	NA	2000	62	7	NA	No	NA	Veterinary Department
Western Bhar Elghazal State, River Jur County	Goats	13/09/2012	NA	6000	200	28	NA	No	NA	Veterinary Department
Eastern Equatoria State, Torit, Iktos Budi, Kapeata North, South and East, and Magwi counties	Goats	20/09/2012	NA	20000	800	30	NA	No	NA	Veterinary Department
Upper Nile State, Renk County	Goats	20/10/2012	NA	NA	NA	NA	NA	No	NA	Veterinary Department
Northern Bhar Elghazal State, Awail East County	Goats	21/10/2012	NA	NA	NA	NA	NA	No	NA	Veterinary Department
Lakes State, Rumbek Centre County	Goats	15/11/2012	NA	NA	NA	NA	NA	No	NA	Veterinary Department
NA	NA	2012	31	NA	NA	NA	NA	NA	NA	[15]
NA	Eland, elephant,	2013	Sero-survey					cELISA		This study

	tiang, white-eared kob									
NA	NA	2013	1	23000	86	36	0	NA	NA	[16]
NA	NA	2014	23	3746	153	95	0	NA	NA	[45]

D.R. of the CONGO

State or region, district or province, (city)	Species	Date	Outbreaks*	S	C	D	S/D	Lab. confirmation	Lineage	Reference
NA	NA	2004	3	NA	NA	NA	NA	NA	NA	[46]
NA	NA	2005	3	NA	77	36	NA	NA	NA	[47]
Western Region, Plateaux Department, Lékana District (Lékana) Djambala District (Djambala, Kahon, and Akou)	Sheep, goats	30/12/2005, 12/04/2006	4	719	614	614	93	cELISA	NA	[48]
NA	NA	2008	1	NA	108	90	17	NA	NA	[12]
NA	NA	2010	1	NA	66	NA	NA	NA	NA	[49]
NA	NA	2011	70	NA	62635	49121	16238	NA	NA	[17]
NA	NA	2012	89	NA	102688	86449	12292	NA	NA	[15] †
Western Region, Kwilu Province	Sheep, goats	23/01/2012,23/06/2014	NA	NA	NA	16250	NA	RT-PCR	NA	[50] †
NA	NA	2013	84	3732743	59742	53872	815	NA	NA	[16] †
NA	NA	2014	67	155187	6298	5812	48	NA	NA	[45] †
Eastern Region, North Kivu Province (Goma)	Sheep	2016	NA	NA	NA	NA	NA	RT-PCR	2	[51]
Eastern Region, North Kivu Province (Kibumba)	Sheep, goats	2017	NA	NA	NA	NA	NA	RT-PCR	3	This study
Western Region, Kinshasa province (N'sele)	Goats	2018	NA	NA	NA	NA	NA	RT-PCR	3	[51]
Eastern Region, South Kivu province (Kalehe)	Goats	2018	NA	NA	NA	NA	NA	RT-PCR	3	[51]

*It is unclear the definition of PPR outbreak in the different reports and publications, the numbers are showed as indicated in each reference.

Northern Region: Napak, Amudat, Kaabong, Moroto, Nakapiripirit, Abim, Kotido, Alebtong, Lira, Pader, Kitgum, Lamwo, Maracha, Moyo, Koboko, Arua, Yumbe, Agago, Otuke Districts. Eastern Region: Bulambuli, Bukedea, Bukwa, Kapchorwa, Kween, Sironko, Mbale, Bugiri, Busia, Jinja Districts; Western Region: Kyegegwa, Kabarole, Isingiro, Rukungiri, Mbarara, Ntungamo Districts; Central Region: Mubende District

†Data for this year reported by AU-IBAR differ from the data at OIE-WAHIS database, both records are included separately.

Table S2. Retrospective information on Peste des petits ruminant antibody detection in wild artiodactyls from natural protected areas in Uganda, tested by cELISA and confirmed with cross neutralization assays (virus neutralization test for Rinderpest virus and Peste des petits ruminant virus deemed positive if ≥2 times titre of contrary assay) under the Pan African Programme for the Control of Epizootics (PACE).

Species	Scientific name	Year	Location	Pos./Sampled	Prev. (CI 95%)	Reference
Buffalo	*Syncerus caffer*	1999	Kidepo Valley National Park	0/11	0.0 (0.0–25.9)	[6,52]
Buffalo	*Syncerus caffer*	1999	Lake Mburo National Park	0/11	0.0 (0.0–25.9)	[6,52]
Impala	*Aepyceros melampus*	1999	Lake Mburo National Park	0/32	0.0 (0.0–10.7)	[6,52]
Buffalo	*Syncerus caffer*	2000	Semuliki National Park	0/10	0.0 (0.0–27.7)	[6,52]
Buffalo	*Syncerus caffer*	2000	Murchison Falls National Park	0/17	0.0 (0.0–18.4)	[6,52]
Buffalo	*Syncerus caffer*	2000	Kibale National Park	0/5	0.0 (0.0–43.4)	[6,52]
Buffalo	*Syncerus caffer*	2000	Pian Upe Wildlife Reserve	0/6	0.0 (0.0–39.0)	[6,52]
Roan antelope	*Hippotragus equinus*	2000	Pian Upe Wildlife Reserve	0/1	0.0 (0.0–94.9)	[6,52]
Hartebeest	*Alcelaphus buselaphus*	2000	Pian Upe Wildlife Reserve	0/2	0.0 (0.0–65.8)	[6,52]
Buffalo	*Syncerus caffer*	2002	Murchison Falls National Park	0/8	0.0 (0.0–32.4)	[6,52]
Buffalo	*Syncerus caffer*	2002	Kidepo Valley National Park	0/17	0.0 (0.0–18.4)	[6,52]
Buffalo	*Syncerus caffer*	2004	Kidepo Valley National Park	2/24	8.3 (2.3–25.8)	Unpublished results
Buffalo	*Syncerus caffer*	2004	Murchison Falls National Park	6/21	28.6 (13.8–50.0)	Unpublished results
Buffalo	*Syncerus caffer*	2005	Queen Elizabeth National Park	8/32	25.0 (13.2–42.1)	Unpublished results

Pos.=positive; Prev.=prevalence

Table S3. Drugs used for wildlife captures in Uganda (buffalo and Uganda kob) and South Sudan (tiang, white eared kob, eland and elephant).

Species		Drug	Dose mg	Company
Buffalo *Syncerus caffer*				
	Sedation	Etorphine hydrochloride	5–10	Novartis Ltd, South Africa
		Xylazine	30–90	Kyron Labs (Pty) Ltd, South Africa
	Reversal	Diprenorphine hydrochloride	16–25	Kyron Labs (Pty) Ltd, South Africa
		Yohimbine hydrochloride	13–26	Kyron Labs (Pty) Ltd, South Africa
Uganda Kob *Kobus kob thomasi*				
	Sedation	azaperone	40–70	Kyron Labs (Pty) Ltd, South Africa
		Haloperidol	15	Kyron Labs (Pty) Ltd, South Africa
	or			
		Etorphine hydrochloride	5	Novartis Ltd, South Africa
		Xylazine	10	Kyron Labs (Pty) Ltd, South Africa
Tiang *Damaliscus lunatus tiang*				
White–eared kob *Kobus kob leucotis*				
	Sedation	Thiafentanil oxalate	5 males<br>3–4 females	Wildlife Pharmaceuticals (Pty) Ltd, South Africa
		Azaperone	60–80 males<br>50 females	Kyron Labs (Pty) Ltd, South Africa
	or			
		Etorphine hydrochloride	4–8 males<br>3–6 females	
		Medetomidine	5 males<br>3–4 females	Pfizer Laboratories (Pty) Ltd, South Africa
	Reversal	Naltrexone hydrochloride	50–150	Wildlife Pharmaceuticals (Pty) Ltd, South Africa
		Atipamezole	5x medetomidine dose	Zoetis (Pty) Ltd, South Africa

Eland *Taurotragus oryx*				
	Sedation	thiafentanil oxalate	15–20	Wildlife Pharmaceuticals (Pty) Ltd, South Africa
		Azaperone	150–200	Kyron Labs (Pty) Ltd, South Africa
	or			
		Etorphine hydrochloride	10–14	Novartis Ltd, South Africa
		Medetomidine	15–20	Pfizer Laboratories (Pty) Ltd, South Africa
	Reversal			
		Naltrexone hydrochloride	100–140	Wildlife Pharmaceuticals (Pty) Ltd, South Africa
		Atipamezole	5x medetomidine dose	Zoetis (Pty) Ltd, South Africa
Elephant *Loxodonta africana*				
	Sedation	Thiafentanil oxalate	12–15	Wildlife Pharmaceuticals (Pty) Ltd, South Africa
		Azaperone	40–60	Kyron Labs (Pty) Ltd, South Africa
	or			
		Etorphine hydrochloride	10–20	Novartis Ltd, South Africa
		Azaperone	40–60	Kyron Labs (Pty) Ltd, South Africa

Table S4. Number of herd owners sampled in western Uganda. When herd owners were not recorded is indicated with NA. The mean number of animals per herd and minimum (min.) and maximum (max.) sizes are indicated by district and species.

District	Subcounty	Cattle			Goats			Sheep		
		2015	2016	2017	2015	2016	2017	2015	2016	2017
**Buliisa district**		0	0	0	NA	0	0	NA	0	0
	Buliisa	0	0	0	NA	0	0	NA	0	0
	Butaba	0	0	0	NA	0	0	0	0	0
Herd mean (min.-max.)			NA			NA			NA	
**Kasese district**		22	NA	14	NA	NA	86	0	NA	17
	Karusandara	3	NA	0	1	0	0	0	0	0
	Katwe-Kabatoro	4	0	4	NA	0	0	0	0	0
	Kisinga	0	0	0	0	0	59	0	0	11
	Kitswamba	0	NA	0	0	NA	0	0	0	0
	Lake Katwe	6	NA	9	NA	NA	0	0	0	0
	Muhokya	4	NA	0	0	NA	0	0	NA	0
	Mukunyu	0	0	0	0	0	27	0	0	6
	Nyakatonzi	5	0	1	0	0	0	0	0	0
Herd mean (min.-max.)			79.0 (15–309)			3.4 (1–25)			1.8 (1–3)	
**Kisoro district**		38	NA	86	NA	NA	92	0	NA	61
	Muramba	34	NA	43	NA	NA	30	NA	NA	34
	Nyarusiza	4	NA	43	NA	NA	62	NA	NA	27
Herd mean (min.-max.)			4.3 (1–17)			4.4 (1–16)			4.2 (1–11)	
**Rubirizi district**		1	NA	0	NA	NA	0	NA	NA	0
	Katanda	0	0	0	NA	0	0	0	0	0
	Kicwamba	0	NA	0	1	NA	0	1	0	0
	Kirugu	0	0	0	NA	0	0	NA	0	0
	Ryeru	1	NA	0	0	NA	0	0	NA	0
Herd mean (min.-max.)			31.0 (2–60)			NA			NA	
**Total**		61	NA	100	NA	NA	178	NA	NA	78

Supplementary material references

1. El Hag Ali, B.; Taylor, W. Isolation of peste des petits ruminants virus from the Sudan. *Res. Vet. Sci.* **1984**, *36*, 1–4.
2. Roeder, P.L.; Abraham, G.; Kenfe, G.; Barretf, T. Peste des Petits Ruminants in Ethiopian Goats. *Trop. Anim. Health Prod.* **1994**, *26*, 69–73.
3. Wamwayi, H.M.; Rossiter, P.B.; Kariuki, D.P.; Wafula, J.S.; Barrett, T.; Anderson, J. Peste des petits ruminants antibodies in East Africa. *Vet. Rec.* **1995**, *136*, 199–200.
4. Banyard, A.C.; Parida, S.; Batten, C.; Oura, C.; Kwiatek, O.; Libeau, G. Global distribution of peste des petits ruminants virus and prospects for improved diagnosis and control. *J. Gen. Virol.* **2010**, *91*, 2885–2897.
5. Khalafalla, A.I.; Saeed, I.K.; Ali, Y.H.; Abdurrahman, M.B.; Kwiatek, O.; Libeau, G.; Obeida, A.A.; Abbas, Z. An outbreak of peste des petits ruminants (PPR) in camels in the Sudan. *Acta Trop.* **2010**, *116*, 161–165.
6. Kock, R.A.; Wamwayi, H.M.; Rossiter, P.B.; Libeau, G.; Wambwa, E.; Okori, J.; Shiferaw, F.S.; Mlengeya, T.D. Re-infection of wildlife populations with rinderpest virus on the periphery of the Somali ecosystem in East Africa. *Prev. Vet. Med.* **2006**, *75*, 63–80.
7. Roeder, P.; Mariner, J.; Kock, R. Rinderpest: The veterinary perspective on eradication. *Philos. Trans. R. Soc. B Biol. Sci.* **2013**, *368*.
8. AU-IBAR *Pan African Animal Health Yearbook 2003*; Interafrican Bureau for Animal Resources and African Union: Nairobi Kenya, 2004; pp. 1–73.
9. Karimuribo, E.D.; Loomu, P.M.; Mellau, L.S.B.; Swai, E.S. Retrospective study on sero-epidemiology of peste des petits ruminants before its official confirmation in northern Tanzania in 2008. *Res. Opin. Anim. Vet. Sci.* **2011**, *1*, 184–187.
10. Luka, P.D.; Erume, J.; Mwiine, F.N.; Ayebazibwe, C. Molecular characterization of peste des petits ruminants virus from the Karamoja region of Uganda (2007-2008). *Arch. Virol.* **2012**, *157*, 29–35.
11. AU-IBAR *Pan African Animal Health Yearbook 2007*; Interafrican Bureau for Animal Resources and African Union: Nairobi, Kenya, 2008; pp. 1–69.
12. AU-IBAR *Pan African Animal Health Yearbook 2008*; Interafrican Bureau for Animal Resources and African Union: Nairobi, Kenya, 2009; pp. 1–81.
13. Ministry of Agriculture Animal Industry and Fisheries MAAIF *Vaccines for the control of neglected animal diseases in Africa (Vacnada).Cumulative Progress Report for December, 2011*; MAAIF: Kamplala, Uganda. pp. 1–30.
14. Muniraju, M.; Munir, M.; Parthiban, A.R.; Banyard, A.C.; Bao, J.; Wang, Z.; Ayebazibwe, C.; Ayelet, G.; Harrak, M. El; Mahapatra, M.; et al. Molecular Evolution of Peste des Petits Ruminants Virus. *Emerg. Infect. Dis.* **2014**, *20*, 2023–2033.
15. AU-IBAR *Pan African Animal Health Yearbook 2012*; Interafrican Bureau for Animal Resources and Afican Union: Nairobi, Kenya, 2013; pp. 1–74.
16. AU-IBAR *Pan African Animal Resources Yearbook 2013*; Interafrican Bureau for Animal Resources and Afican Union: Nairobi, Kenya, 2014; pp. 1–107.
17. AU-IBAR *Panafrican Animal Health Yearbook 2011*; Interafrican Bureau for Animal Resources and Afican Union: Nairobi, Kenya, 2012; pp. 1–92.
18. Nakayima, J.; Nanfuka, M.; Kidega, E.; Kajuna, Y.; Ndumu, D. Epidemiological Insights into the Occurrence of Peste des Petits Ruminants Virus (PPRV) Among Sheep, Goats and Cattle in Western Uganda. *Int. J. Livest. Res.* **2018**, *8*, 73.
19. Sande, R.; Ayebazibwe, C.; Waiswa, C.; Ejobi, F.; Mwiine, F.N.; Olaho-Mukani, W. Evidence of Peste Des Petits Ruminants Virus Antibodies in Small Ruminants in Amuru and Gulu Districts, Uganda. *Pak. Vet. J.* **2011**, *31*, 363–365.

20. Waret-szkuta, A.; Roger, F.; Chavernac, D.; Yigezu, L.; Libeau, G.; Pfeiffer, D.U.; Guitián, J. Peste des Petits Ruminants (PPR) in Ethiopia: Analysis of a national serological survey. *BMC Vet. Res.* **2008**, *4*, 1–10.
21. Torsson, E.; Kgotlele, T.; Berg, M.; Mtui-Malamsha, N.; Swai, E.S.; Wensman, J.J.; Misinzo, G. History and current status of peste des petits ruminants virus in Tanzania. *Infect. Ecol. Epidemiol.* **2016**, *6*, 1–7.
22. Nkamwesiga, J.; Coffin-Schmitt, J.; Ochwo, S.; Mwiine, F.N.; Palopoli, A.; Ndekezi, C.; Isingoma, E.; Nantima, N.; Nsamba, P.; Adiba, R.; et al. Identification of peste des petits ruminants transmission hotspots in the Karamoja subregion of Uganda for targeting of eradication interventions. *Front. Vet. Sci.* **2019**, *6*, 1–13.
23. Dundon, W.G.; Kihu, S.M.; Gitao, G.C.; Bebora, L.C.; John, N.M.; Oyugi, J.O.; Loitsch, A.; Diallo, A. Detection and Genome Analysis of a Lineage III Peste Des Petits Ruminants Virus in Kenya in 2011. *Transbound. Emerg. Dis.* **2015**, *64*, 644–650.
24. Kihu, S.M.; Gachohi, J.M.; Ndungu, E.K.; Gitao, G.C.; Bebora, L.C.; John, N.M.; Wairire, G.G.; Maingi, N.; Wahome, R.G.; Ireri, R. Sero-epidemiology of Peste des petits ruminants virus infection in Turkana County, Kenya. *BMC Vet. Res.* **2015**, *11*, 1–13.
25. Olupot, W.; Mcneilage, A.J.; Plumptre, A.J. *An analysis of socioeconomics of bushmeat hunting at major hunting sites in Uganda*; 2009;
26. Plumptre, A.J.; Nangendo, G.; Ayebare, S.; Kirunda, B.; Mugabe, H.; Nsubuga, P. & Nampindo, S. Impacts of climate Change and Industrial Development on the long-term changes in Wildlife Behavior in the Greater Virunga Landscape. *Wildl. Conserv. Soc.* **2017**.
27. Floody, O.R.; Arnold, A.P. Uganda Kob (*Adenota kob thomasi*): territoriality and the spatial distributions of sexual and agonsitic behaviours at a territorial group. *Z. Tierpsychol.* **1975**, *37*, 192–212.
28. Plumptre, A.J.; Kujirakwinja, D.; Moyer, D.; Driciru, M.; Rwetsiba, A. *Greater Virunga Landscape, Large mammal survey 2010*; Uganda Wildlife Authority (UWA): Kampala, Uganda, 2010; pp 1–15.
29. Woodford, M.H. Tuberculosis in wildlife in the Ruwenzori National Park, Uganda (Part I). *Trop. Anim. Health Prod.* **1982**, *14*, 81–88.
30. UBOS *The National Population and Housing Census 2014 – Main Report*; Kampala, Uganda, 2016;
31. MAAIF and UBOS The national livestock census. A summary report of the national livestock census 2008. *Uganda Bur. Stat.* **2009**, *May*, 1–34.
32. Meunier, N. V.; Sebulime, P.; White, R.G.; Kock, R. Wildlife-livestock interactions and risk areas for cross-species spread of bovine tuberculosis. *Onderstepoort J. Vet. Res.* **2017**, *84*, 1–10.
33. Fay, M.; Elkan, P.; Marjan, M.; Grossmann, F. *Aerial Surveys of Wildlife, Livestock, and Human Activity in and around Existing and Proposed Protected Areas of Southern Sudan, Dry Season 2007. Phase I.*; Juba, 2007;
34. GOSS; SSCCSE *Southern Sudan Counts: Tables from the 5th Sudan Population and Housing Census, 2008*; 2010;
35. National Bureau of Statistics *Population projections for South Sudan by County 2015-2020*; 2015;
36. Plumptre, A.J.; Davenport, T.R.B.; Behangana, M.; Kityo, R.; Eilu, G.; Ssegawa, P.; Ewango, C.; Meirte, D.; Kahindo, C.; Herremans, M.; et al. The biodiversity of the Albertine Rift. *Biol. Conserv.* **2007**, *134*, 178–194.
37. CTA/Université Evangélique en Afrique *Building the Evidence Base on the Agricultural Nutrition Nexus: Democratic Republic of Congo*; Agriculture and nutrition; 2015;
38. Büscher, K.; Mathys, G. War, Displacement and Rural–Urban Transformation: Kivu's Boomtowns, Eastern D.R. Congo. *Eur. J. Dev. Res.* **2019**, *31*, 53–71.
39. AU-IBAR *Pan African Animal Health Yearbook 2006*; Interafrican Bureau for Animal Resources and Afican Union: Nairobi, Kenya, 2007; pp. 1–85.

40. AU-IBAR *Pan African Animal Health Yearbook 2007*; Interafrican Bureau for Animal Resources and Afican Union: Nairoby, Kenya, 2008; pp. 1–69.
41. OIE-WAHIS. The World Animal Health Information System, Peste des petits Ruminants, Uganda. Available online: https://www.oie.int/wahis_2/public/wahid.php/Reviewreport/Review?page_refer=MapFullEventReport&reportid=5837.
42. Ruhweza, S.P.; Ayebazibwe, C.; Mwiine, F.N.; Muhanguzi, D.; Mulindwa, D.; Olaho-Mukani, W. Seroprevalence of Peste des Petits Ruminants (PPR) virus antibodies in goats and sheeps in North-Eastern Uganda. *Bull. Anim. Heal. Prod. Africa* **2010**, 141–146.
43. Mulindwa, B.; Ruhweza, S.P.; Ayebazibwe, C.; Mwiine, F.N.; Muhanguzi, D.; Olaho-mukani, W. Peste des Petits Ruminants serological survey in Karamoja sub region of Uganda by competitive ELISA. *Vet. World* **2011**, *4*, 149–152.
44. Lernfelt, L. Prevalence of antibodies for Peste des petits des ruminants virus and Brucella and related risk factors in goat herds in urban and peri-urban agriculture in Kampala, Uganda, Second cycle thesis, A2E. Sveriges lantbruksuniversitet (SLU), Uppsala, Sweden, 2013.
45. AU-IBAR *Pan African Animal Health Yearbook 2014*; Interafrican Bureau for Animal Resources and Afican Union: Nairobi, Kenya, 2015; pp. 1–148.
46. AU-IBAR *Pan African Animal Health Yearbook 2004*; Interafrican Bureau for Animal Resources and Afican Union: Nairobi, Kenya, 2005; pp. 1–73.
47. AU-IBAR *Pan African Animal Health Yearbook 2005*; Interafrican Bureau for Animal Resources and Afican Union: Nairobi, Kenya, 2006; pp. 1–85.
48. OIE-WAHIS. The World Animal Health Information System, Peste des petits ruminants, Congo (Rep. of the). Available online: https://www.oie.int/wahis_2/public/wahid.php/Reviewreport/Review?page_refer=MapFullEventReport&reportid=5412.
49. AU-IBAR *Pan African Animal Health Yearbook 2010*; Interafrican Bureau for Animal Resources and Afican Union: Nairobi, Kenya, 2011; pp. 1–104.
50. OIE-WAHIS. The World Animal Health Information System, Peste des petits ruminants, Congo (Rep. of the). Available online: https://www.oie.int/wahis_2/public/wahid.php/Reviewreport/Review?reportid=11810.
51. Tshilenge, M.G.; Walandila, J.S.; Byakya, D.; Masumu, J.; Katshay, L.; Cattoli, G.; Bushu, E.; Mpiana, S.; Dundon, W.G. Peste des petits ruminants viruses of lineages II and III identified in the Democratic Republic of the Congo. *Vet. Microbiol.* **2019**, *239*, 108493.
52. Kock, R.A. The Role of Wildlife in the Epidemiology of Rinderpest in East and Central Africa 1994-2004: A Study Based on Serological Surveillance and Disease Investigation. Thesis for the Degree of Doctor of Veterinary Medicine., University of Cambridge, Cambridge, England, 2008.
